# 2-Methylimidazole Copper Iminodiacetates for the Adsorption of Oxygen and Catalytic Oxidation of Cyclohexane

**DOI:** 10.3390/molecules25061286

**Published:** 2020-03-12

**Authors:** Xi Chen, Dong-Li An, Xin-Qi Zhan, Zhao-Hui Zhou

**Affiliations:** 1 State Key Laboratory for Physical Chemistry of Solid Surfaces, College of Chemistry and Chemical Engineering, Xiamen University, Xiamen 361005, China; xichen@stu.xmu.edu.cn (X.C.); andongli@xmu.edu.cn (D.-L.A.); 2 Medical College, Xiamen University, Xiamen 361005, China; zxq@xmu.edu.cn

**Keywords:** copper, iminodiacetic acid, 2-methylimidazole, catalytic oxidation of cyclohexane, gas adsorption

## Abstract

The mixed-ligand copper(II) iminodiacetates [Cu(ida)(2-mim)(H_2_O)_2_]·H_2_O (**1**), [Cu(ida)(2-mim)_2_]·2H_2_O (**2**), [Cu(ida)(2-mim)(H_2_O)]_n_·4.5nH_2_O (**3**), and [Cu_2_(ida)_2_(2-mim)_2_]_n_·nH_2_O (**4**) (H_2_ida = iminodiacetic acid, 2-mim = 2-methylimidazole) were obtained from neutral or alkaline solutions at different temperatures. The novel complex **4** contains very small holes with diameters of 2.9 Å, which can adsorb O_2_ selectively and reversibly between 1.89 to 29.90 bars, compared with the different gases of N_2_, H_2_, CO_2_, and CH_4_. This complex is stable up to 150 °C based on thermal analyses and XRD patterns. The four complexes show catalytic activities that facilitate the conversion of cyclohexane to cyclohexanol and cyclohexanone with hydrogen peroxide in a solution. The total conversion is 31% for **4**.

## 1. Introduction

Copper is a trace metal element that is essential for life and is commonly found in plants and animals in the form of coordination compounds [1]. The rational design and synthesis of novel mixed-ligand copper complexes are still in progress [2,3,4,5,6]. Because of their favorable combination of redox properties in coordination chemistry, copper complexes play essential roles in catalytic reactions [7,8,9]. The selective oxidation of cyclohexane has industrial and economic importance, considering the significance of its oxidized products (cyclohexanol and cyclohexanone) in the preparation of adipic acid and caprolactam as precursors of Nylon-6,6 and polyamides. Owing to the intrinsic inertness of C–H bonds, harsh reaction conditions are often required at high temperatures and pressures [10], while particulate methane monooxogenase (pMMO), an enzyme that bears a multicopper cluster with an N,O-environment, constitutes a paradigmatic example of a catalyst for selective C–H oxidation under mild conditions [11,12]. Some monomeric, dimeric, trimeric, and tetrameric copper complexes have been tested as catalysts for the oxidative reaction of cyclohexane [13,14,15,16,17,18,19]. The type of ligand and the configuration of the complexes play crucial roles in the catalytic performance of copper complexes. Aminopolycarboxylic ligands, such as iminodiacetic acid, are not only strong chelate ligands but also feature the coordination diversity to form coordination polymers in different synthetic conditions [20,21]. Imidazole ligands are also widely used in the syntheses of metal complexes due to their coordination abilities [22]. For achieving an N, O environment around Cu centers, we selected iminodiacetic acid as a chelating ligand and 2-methylimidazole as the second ligand, in order to synthesize complexes with different configurations under different reaction conditions.

In this study, the mixed-ligand copper(II) complexes [Cu(ida)(2-mim)(H_2_O)_2_]·H_2_O (**1**), [Cu(ida)(2-mim)_2_]·2H_2_O (**2**), [Cu(ida)(2-mim)(H_2_O)]_n_·4.5nH_2_O (**3**), and [Cu_2_(ida)_2_(2-mim)_2_]_n_·nH_2_O (**4**) (H_2_ida = iminodiacetic acid, 2-mim = 2-methylimidazole) were isolated at different temperatures. There are 2.9 Å holes in **4** that can adsorb O_2_ selectively at room temperature compared with different gas adsorption types at different pressures. The catalytic performance of copper iminodiacetates in the oxidation of cyclohexane was tested and discussed.

## 2. Results and Discussion

### 2.1. Syntheses

**Scheme 1:**
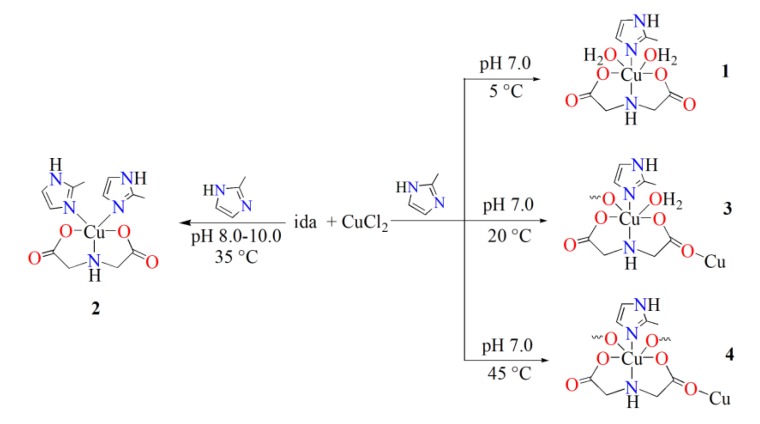
Synthetic conditions for 2-methylimidazole copper iminodiacetates **1**–**4**.

The syntheses of 2-methylimidazole copper iminodiacetates show pH- and temperature-dependent modes. The pH value may affect the coordination ability of 2-methylimidazole, as well as the temperature for the formation of monomer **1** and coordination polymers **3** and **4**. In the reaction of the copper iminodiacetate with 2-methylimidazole at pH 7.0, monomeric copper iminodiacetate [Cu(ida)(2-mim)(H_2_O)_2_]·H_2_O (**1**) was isolated at 5 °C. As the temperature increased, one water molecule was substituted by iminodiacetate, resulting in the formation of a one dimensional coordination polymer [Cu(ida)(2-mim)(H_2_O)]_n_·4.5nH_2_O (**3**) at 20 °C. With an increase of temperature, the arrangement of the [Cu(ida)(2-mim)] unit changed. The [Cu(ida)(2-mim)] unit can coordinate two adjacent structural units at the same time, thus yielding the final product [Cu_2_(ida)_2_(2-mim)_2_]_n_·nH_2_O (**4**) at 45 °C as shown in Scheme 1. Alkaline conditions enhanced the coordination ability of 2-methylimidazole, resulting in the formation of [Cu(ida)(2-mim)_2_]·2H_2_O (**2**) between pH 8.0 and 10.0.

### 2.2. Crystal Structures

**Figure 1:**
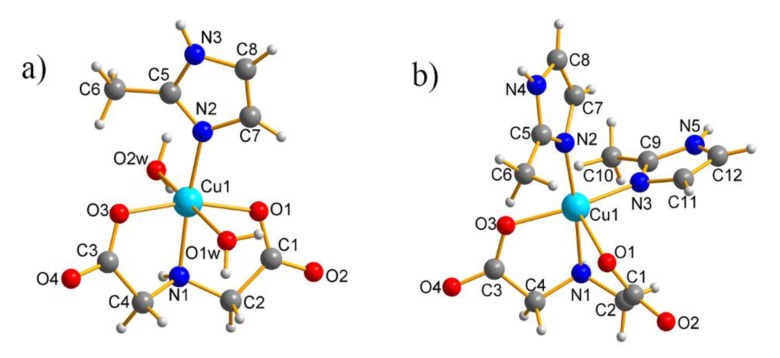
(**a**) Plot of the molecular structure in [Cu(ida)(2-mim)(H_2_O)_2_]·H_2_O (**1**). (**b**) Plot of the molecular structure in [Cu(ida)(2-mim)_2_]·2H_2_O (**2**).

Detailed crystallographic data are given in Table 1. The packing diagrams of **1**–**4** are shown in Figures S1–S4. Monomer [Cu(ida)(2-mim)(H_2_O)_2_] (**1**) contains a Cu ion in a distorted octahedral N_2_O_4_ environment. In the crystal structure, the Cu ion is surrounded by two water molecules, one 2-methylimidazole, and two chelate rings via amine nitrogen and oxygen atoms from the iminodiacetate as shown in Figure 1a. The bond lengths are within the range of 1.955 and 2.755 Å. The free water molecule O3w forms hydrogen bonds with the iminodiacetate ligands [O3w···O1 2.743(3), O3w···O1wc 2.759(3) Å, c (1 + *x*, *y*, *z*)], which can stabilize the main structure of **1**. The detailed hydrogen bonds are listed in Table S2.

The molecular structure of **2** is shown in Figure 1b. The Cu ion is coordinated by one tridentate iminodiacetate dianion and two 2-methylimidazole molecules in a distorted tetragonal pyramidal coordination environment. Two nitrogen atoms from 2-methylimidazole and one oxygen atom from iminodiacetate are positioned in the bottom plane, and the other oxygen atom of iminodiacetate is at the apical position. Because of the Jahn–Teller effect, the bond length of Cu−O(1) is elongated to 2.300(2) Å.

**Figure 2:**
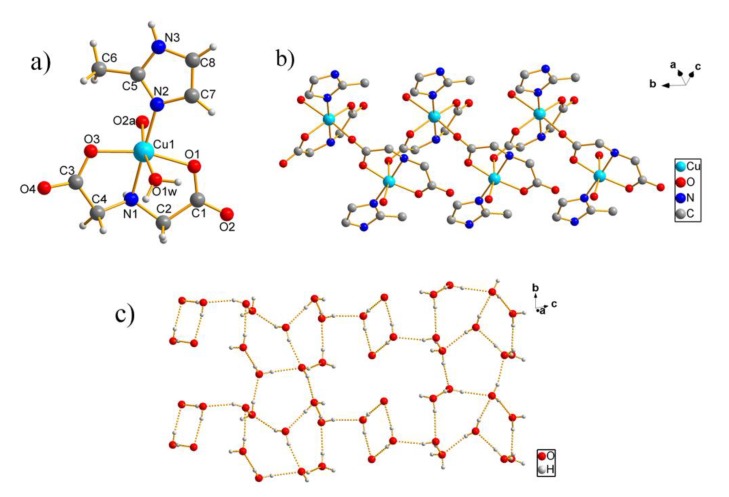
(**a**) Plot of the molecular structure in [Cu(ida)(2-mim)(H_2_O)]_n_·4.5nH_2_O (**3**). (**b**) The chain structure in [Cu(ida)(2-mim)(H_2_O)]_n_·4.5nH_2_O (**3**); hydrogen atoms are omitted for clarity. (**c**) Water clusters in [Cu(ida)(2-mim)(H_2_O)]_n_·4.5nH_2_O (**3**).

An x-ray structural analysis revealed that **3** is a chain coordination polymer with a neutral unit Cu(ida)(2-mim) and one coordinated water molecule. As shown in Figure 2a, the Cu ion exists in an elongated N_2_O_4_ octahedral coordination environment, which contains three carboxy oxygen atoms, two nitrogen atoms, and one water molecule. Because of the Jahn–Teller effect, the axial positions are weakly coordinated by one carboxy oxygen atom [2.460(2) Å] and one water molecule [2.501(2) Å]. Ida uses its two carboxy oxygen atoms and one nitrogen atom to chelate with a copper ion and still uses its other carboxy oxygen atoms to coordinate with an adjacent copper ion, forming a chain structure as shown in Figure 2b.

The striking feature in **3** is the formation of water layers as shown in Figure 2c. The formation of the water layer indicates that hydrogen bonds play crucial roles in stabilizing the host structure. In **3**, the water molecules and oxygen atoms in iminodiacetates connect to each other by hydrogen bonds, forming a series of cyclic tetramer, pentamer, and hexamer water clusters. The water cluster rings form an infinitely extending 2D network structure. The water layers connect and fill the vacant portion between the two molecular layers of **3**. The average O···O length of the water layer is 2.780(3) Å, which is longer than the corresponding value in ice *I*_h_ (2.759 Å) [23] and shorter than that in liquid water (2.854 Å) [24] but within the values in the ice II phase (2.77–2.84 Å) [25]. The identification and characterization of the water clusters in various crystalline materials can help us understand the properties of bulk water in both its liquid and solid states, as well as the water–water interactions in various environments [26,27,28]. The hydrogen bonds are listed in Table S4.

**Figure 3:**
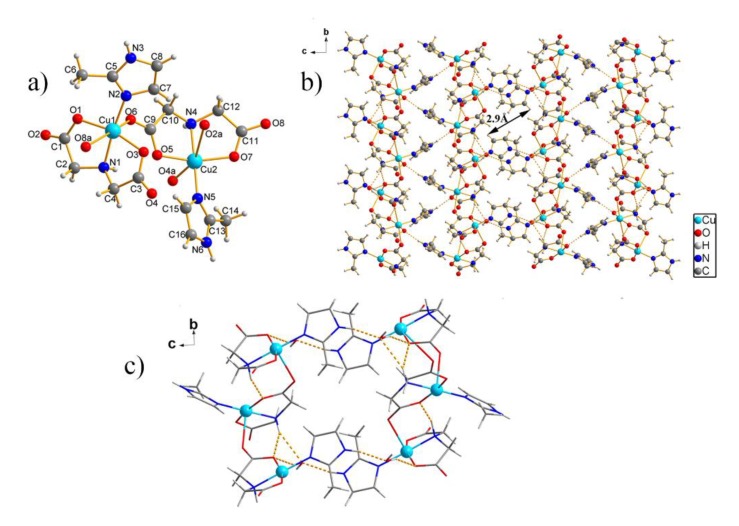
(**a**) Plot of the molecular structure in [Cu_2_(ida)_2_(2-mim)_2_]_n_·nH_2_O (**4**). (**b**) Structure of the holes in [Cu_2_(ida)_2_(2-mim)_2_]_n_·nH_2_O (**4**) viewed along the *a* axis. (**c**) The smallest constituent unit of the channel (minimum inner diameters = 2.9 Å) in [Cu_2_(ida)_2_(2-mim)_2_]_n_·nH_2_O (**4**). Color codes: sky blue for Cu ions.

The x-ray structural analysis revealed that **4** is a two dimensional coordination polymer with a dinuclear unit Cu_2_(ida)_2_(2-mim)_2_ and water molecules. As shown in Figure 3a, the coordination site of the water molecule in **3** is further displaced by a carboxy oxygen atom of iminodiacetate. The Cu ion exists in an elongated N_2_O_4_ octahedral coordination environment, which contains four carboxy oxygen atoms and two nitrogen atoms. Because of the Jahn–Teller effect, the axial positions are weakly coordinated by two carboxy oxygen atoms [2.350(3) Å and 2.606(3) Å]. The iminodiacetate in **4** uses its two carboxy oxygen atoms and one nitrogen atom to chelate one copper ion (Cu1) and still uses its two other carboxy oxygen atoms to coordinate with two adjacent copper ions (Cu2), forming a 2D porous structure. The hydrogen bonds of the water molecules play important roles in the crystal packing of the structure (Table S5). The O_carboxy_···O_water_ and N···O_water_ lengths are in the range of 2.773(5) to 3.190(5) Å and show strong hydrogen bonding among the water molecules, 2-methylimidazoles, and iminodiacetates.

There are small holes in the structure of **4** viewed along the *a* axis as shown in Figure 3b. The channel exhibits a regular rectangle shape. The minimum inner diameters of the channels are 2.9 Å. The smallest constituent unit of the channel includes 6 Cu ions. If [Cu(ida)(2-mim)] is considered as the node for topology purposes, the networks of **4** can be simplified into a 4-connected uninodal network with the Schläfli symbol of (4^4^·6^2^) as shown in Figure S5.

Comparisons of the selected bond lengths (Å) for **1**–**4** and the other iminodiacetate copper complexes are shown in Tables S1 and S6–S9. The Cu–O lengths in planes **1** and **2** [1.985(2)_av_ Å, 1.962(2) Å] are similar to those of the other copper iminodiacetates [1.953(2)_av_–1.990(1) Å] [29,30,31,32,33,34]. However, in **3** and **4**, the Cu–O lengths in plane [1.997(2)_av_ Å, 1.991(3)_av_ Å] are slightly longer than the other copper iminodiacetates. Because of Jahn–Teller effect, the Cu–O lengths in the axial positions [2.405_av_ Å] are longer than the Cu–O lengths in the plane [1.976_av_ Å]. The Cu–O lengths in the axial positions in **1** [2.580_av_ Å] are longer than the other copper iminodiacetates. Moreover, the bond lengths of Cu–N_ida_ in **1**–**4** [1.998(2) Å, 2.042(2) Å, 1.991(2) Å, and 1.974(3) Å] are longer than those of Cu–N_2-mim_ [1.955(2) Å, 1.985(2)_av_ Å, 1.953(2) Å, and 1.965(3) Å], which implies that the bonds of Cu–N_2-mim_ are stronger.

The theoretical bond valence calculations [35] (Table S10) give the valences of 2.106 for **1**, 2.181 for **2**, 2.179 for **3**, 2.213, and 2.082 for **4**, which together indicate that all Cu ions are in a +2 oxidation state for **1**–**4**.

### 2.3. IR Spectra

The IR spectra for **1**–**4** are presented in Figure S6. The bands of **1**–**4** at 1633–1566 cm^−1^ and 1402–1326 cm^−1^ are attributed to the asymmetric and symmetric vibrations of the carboxy groups of iminodiacetate ligands. Compared with free carboxylic acidic groups (1740–1700 cm^−1^), the absorption peaks are red shifted due to the coordination of carboxy groups with Cu ions, which reduce the bond strength of C−O bonds. The strong symmetrical vibration peaks of the carboxy groups of monomers **1** and **2** appear at 1396 cm^−1^ and 1402 cm^−1^, while those of polymers **3** and **4** appear at lower wave numbers (1377 cm^−1^ and 1375 cm^−1^). This may be attributable to the coordination modes for bridging the carboxy groups in the polymers.

### 2.4. Thermogravimetricanalyses (TGA)

The TGA curves of polycrystalline samples **1**–**3** and the derivatives of mass loss are shown in Figures S7–S9. The first large weight losses of **1** and **2** from 71 to 140 °C correspond to the losses of the crystallized and coordination water molecules, which lose about 16.38 wt.% and 9.40 wt.%, respectively. The water mass losses are consistent with the theoretical calculations (16.31% for **1** and 9.12% for **2**). The water mass loss of **3** (14.72%) is less than the theoretical calculation (26.34%) because **3** tends to lose water at room temperature, and parts of the water molecules were lost before the thermogravimetric analysis. The weight losses at 223 °C and 280 °C for **1**; 196 °C, 222 °C, and 195 °C for **2**; and 226 °C and 268 °C for **3** can be assigned to the decomposition of the iminodiacetate and 2-methylimidazole ligands. The residues of **1**–**3** are copper oxide.

**Figure 4:**
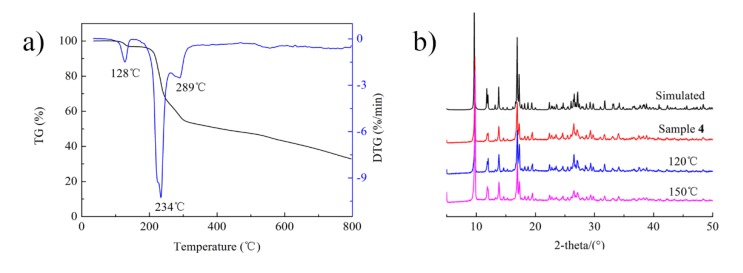
(**a**) TG-DTG curves of [Cu_2_(ida)_2_(2-mim)_2_]_n_·nH_2_O (**4**). (**b**) XRD patterns of [Cu_2_(ida)_2_(2-mim)_2_]_n_·nH_2_O (**4**) at different temperatures.

The TGA curves of [Cu_2_(ida)_2_(2-mim)_2_]_n_·nH_2_O (**4**) are shown in Figure 4a. The first weight loss at 128 °C corresponds to the loss of the water molecules. Then, the structure of **4** seems to be stable up to 150 °C. Sample **4** was further heated at 120 °C and 150 °C for one hour and characterized by its XRD patterns. As shown in Figure 4b, the pattern of **4** was consistent with that of the simulation, showing that the sample contains no impurities. The diffraction patterns of the heated samples remained unchanged, which implies that the 2D framework did not break up and remained stable after losing water molecules.

### 2.5. Gas Adsorption

**Figure 5:**
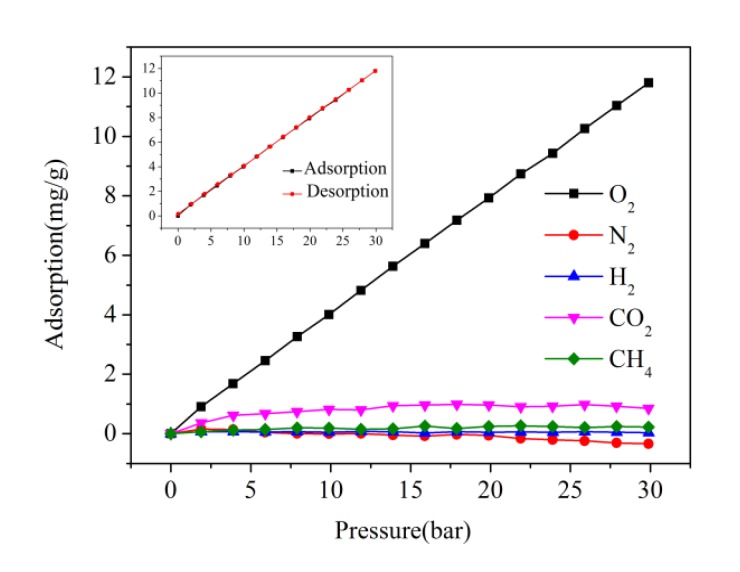
Adsorption isotherms of [Cu_2_(ida)_2_(2-mim)_2_]_n_·nH_2_O (**4**) for the gases of O_2_, N_2_, H_2_, CO_2_, and CH_4_ at 298 K under different pressures; the upper left corner of the picture shows the adsorption and desorption behaviour of O_2_ under the same conditions.

Porous material **4** was synthesized in large quantities for the adsorption of gases. Compared with the absorption of the five common gas types N_2_, O_2_, CO_2_, CH_4_, and H_2_ under the same conditions, we found that **4** can adsorb O_2_ selectively. The maximum adsorption is 12 mg/g at 30 bar O_2_ with a low extent. As the pressure rises, the amount of O_2_ adsorbed gradually increases from 0.91, 3.27, 6.40, and 11.80 mg/g at 1.9, 7.9, 15.9, and 29.9 bar, respectively. However, the other four gases experience only very little or even no adsorption, which can be seen in Figure 5. Detailed adsorption data are given in Table S11. These results suggest that **4** possesses the properties of the identification and separation of O_2_ from the other gases of N_2_, CO_2_, H_2,_ and CH_4_. In addition, we added the adsorption and desorption experiments for oxygen under the same conditions, which can be seen in the left corner of Figure 5. We found that the adsorption and the desorption curves are almost identical, showing that the adsorption process of oxygen is reversible. The effective dimensions of the channels in **4** are approximately 2.9 Å in diameter. The selective gas adsorption in **4** seems to be related to both the pore size and the interactions between the adsorbed molecules and pore walls. Moreover, there are many oxyphilic groups, like carboxy groups, among the iminodiacetic ligands in the pores that can interact with O_2_ more strongly than the other gases.

### 2.6. Catalytic Performance for the Oxidation of Cyclohexane

As shown in Table 2, all of four complexes were used as catalytic precursors for the oxidation of cyclohexane to cyclohexanol and cyclohexanone with hydrogen peroxide in acetonitrile. A blank test confirmed that no cyclohexane oxidation proceeded in the absence of copper catalyst precursors. It should also be noted that the catalytic precursors did not remain intact over the course of catalytic experiments. Upon treatment with H_2_O_2_ and an acid promoter, the monocopper catalytic active species [Cu(ida)(2-mim)] for **1** and **3**, [Cu(ida)(2-mim)_2_] for **2**, and the bicopper catalytic active species [Cu_2_(ida)_2_(2-mim)_2_] for **4** were generated [15,36,37]: **1** has a yield up to 22%, which is close to that of polymer **3**. This is attributed to the weak coordination of the axial water molecules or oxygen atoms. In the catalytic reactions, these atoms may separate from the copper atoms to form unsaturated copper centers, resulting in the formation of mono copper catalytic active species [Cu(ida)(2-mim)]. Compared with **1**, **2** can only achieve a yield of 18%, which may be attributable to the steric hindrance of 2-methylimidazole. Moreover, the overall yield increased to 31% for the dinuclear complex [Cu_2_(ida)_2_(2-mim)_2_] derived from **4** under catalytic conditions. The greatest catalytic activity is assigned to the binuclear copper active center. A recent catalytic report for the copper complexes of functionalized 2,2′:6′,2″-terpyridines and 2,6-di(thiazol-2-yl) pyridine showed a 23% yield [38]. The copper coordination polymer [{Cu(bea)(Hbea)}_4_(μ_4_-pma)]_n_·2nH_2_O (Hbea = N-benzylethanolamine, H_4_pma = pyromellitic acid) can also be used as a catalyst for cyclohexane oxidation, and the conversion rate was 33% [15]. The best copper catalyst [Cu/(*R,R*)-BPBP]^+^ ((*R,R*)-BPBP = (*2R,2′R*)-1,1′-bis(2-pyridylmethyl)-2,2′-bipyrrolidine) achieved excellent catalytic performance with a yield of 56% [39]. Although the catalytic performance of **1**–**4** was inferior, these yields can be considered good due to the high inertness of the saturated hydrocarbons and the mild reaction conditions [40,41].

**Scheme 2:**
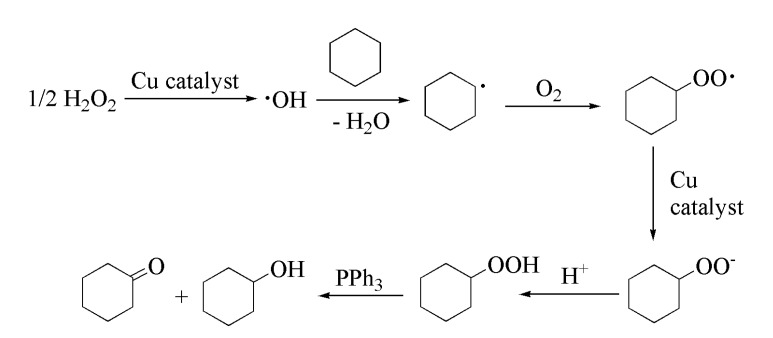
Proposed mechanistic pathway for the oxidation of cyclohexane.

The proposed reaction mechanism (Scheme 2) involves the participation of hydroxyl radicals as principal oxidizing species. These radicals are formed when hydrogen peroxide reacts with a copper catalyst and the abstract hydrogen atoms of cyclohexane to produce cyclohexane radicals. Then, the radicals react with dioxygen (present in the air or generated from H_2_O_2_) to form CyOO· (cyclohexaneperoxy radicals), which are converted to CyOOH products (cyclohexane hydroperoxides) in an acidic solution. After being treated with triphenylphosphine (PPh_3_), the CyOOH product decomposes to its final oxidation products, cyclohexanol and cyclohexanone. However, the detailed mechanism of this process is unclear.

## 3. Materials and Methods

### 3.1. Materials and Instrumentation

The chemicals used were analytical grade reagents. The pH values were determined by a PHB-8 digital pH meter. The infrared spectra were recorded as Nujol mulls between KBr plates on a Nicolet 380 FT-IR spectrometer (Waltham, MA, USA). Elemental analyses were performed with a Vario ELIII elemental analyzer (Hanau, Hessen, Germany). The solution ^1^H NMR spectra for the reaction mixtures were recorded on a Bruker AV 400 NMR spectrometer (Fallanden, Zurich, Switzerland) with CDCl_3_ containing 0.03% TMS (tetramethylsilane) as an internal reference. TG analyses were performed on a Netzsch TG209F1 instrument (Selb, Bavaria, Germany) in N_2_ at a heating rate of 10 °C min^−1^. The gas adsorption capacities of **4** were evaluated with a magnetic suspension gravimetric sorption analyzer (ISOSORP-HTGRA, New Castle, DE, USA) at 298 K at different pressures.

### 3.2. Preparation of [Cu(ida)(2-mim)(H_2_O)_2_]·H_2_O (**1**)

The CuCl_2_·2H_2_O (170.5 mg, 1.0 mmol), H_2_ida (133.1 mg, 1.0 mmol), and 2-methylimidazole (82.1 mg, 1.0 mmol) were dissolved in water (10.0 mL). The pH value of the mixture was adjusted to 7.0 with the addition of KOH under continuous stirring and left standing at 5 °C for one week to yield a blue crystalline solid of **1.** The yield was 64% (213.0 mg) based on the copper chloride. The analytical calculations for C_8_H_17_CuN_3_O_7_: C, 29.1; H, 5.3; N, 12.9 found: C, 29.0; H, 5.2; N, 12.7 (%); IR (KBr, cm^−1^): 3432_vs_, 3271_s_, 3136_m_, 2923_m_, 1629_vs_, 1495_w_, 1438_w_, 1421_w_, 1396_s_, 1382_m_, 1301_w_, 1119_w_, 1021_w_, 764_m_, 594_w_.

### 3.3. Preparation of [Cu(ida)(2-mim)_2_]·2H_2_O (**2**)

The CuCl_2_·2H_2_O (170.5 mg, 1.0 mmol), H_2_ida (133.1 mg, 1.0 mmol), and 2-methylimidazole (81.2 mg, 1.0 mmol) were dissolved in water (10.0 mL). The pH value of the mixture was adjusted to 8.0–10.0 with the addition of KOH under continuous stirring and left standing at 35 °C. Product **2** was isolated as blue blocks after two weeks. The yield was 73% (290.0 mg) based on copper chloride. Calculations for C_12_H_21_CuN_5_O_6_: C, 36.6; H, 5.5; N, 17.6 found: C, 36.5; H, 5.4; N, 17.7 (%); IR (KBr, cm^−1^): 3500_vs_, 3420_s_, 3230_s_, 3124_m_, 3078_m_, 2918_m_, 1660_m_, 1598_vs_, 1566_s_, 1438_m_, 1402_s_, 1382_m_, 1297_m_, 1135_m_, 1118_m_, 765_m_.

### 3.4. Preparation of [Cu(ida)(2-mim)(H_2_O)]_n_·4.5nH_2_O (**3**)

CuCl_2_·2H_2_O (170.5 mg, 1.0 mmol), H_2_ida (133.1 mg, 1.0 mmol), and 2-methylimidazole (82.1 mg, 1.0 mmol) were dissolved in water (10.0 mL). The pH value of the mixture was adjusted to 7.0 with the addition of KOH under continuous stirring and left standing at 20 °C for one week to give a blue crystalline solid of **3**. The yield was 46% (173.0 mg) based on copper chloride. Calculations for C_8_H_22_CuN_3_O_9.5_: C, 25.5; H, 5.8; N, 11.3 found: C, 25.7; H, 5.9; N, 11.2 (%); IR (KBr, cm^−1^): 3432_vs_, 3271_m_, 3111_m_, 2921 _m_, 1632 _vs_, 1575 _m_, 1377_s_ 1321_w_, 1134_m_, 1111_m_, 907_m_, 761_w_, 751_m_.

### 3.5. Preparation of [Cu_2_(ida)_2_(2-mim)_2_]_n_·nH_2_O (**4**)

CuCl_2_·2H_2_O (170.5 mg, 1.0 mmol), H_2_ida (133.1 mg, 1.0 mmol), and 2-methylimidazole (82.1 mg, 1.0 mmol) were dissolved in water (10.0 mL). The pH value of the mixture was adjusted to 7.0 with the addition of KOH under continuous stirring. The solution was slowly evaporated at 45 °C and product **4** was isolated as blue plates after one week. The yield was 31% (178.5 mg) based on copper chloride. Calculations for C_16_H_24_Cu_2_N_6_O_9_: C, 33.5; H, 4.3; N, 14.5 found: C, 33.6; H, 4.2; N, 14.7(%); IR (KBr, cm^−1^): 3424_vs_, 3272_m_, 3110_m_, 3046_w_, 2921_m_, 1633_vs_, 1575_w_, 1375_s_, 1321_w_, 1135_m_, 1110_m_, 906_m_, 761_w_, 751_m_.

### 3.6. X-ray Structure Determinations

Crystals **1**–**4** were measured on an Oxford Gemini CCD diffractometer with graphite monochromated Mo Kα radiation (λ = 0.71073 Å) at 193 K. The structures were primarily solved by Olex2 and ShelXT in the WinGX program [42] and refined by full-matrix least-squares procedures with anisotropic thermal parameters for all of the nonhydrogen atoms with SHELX-2018/3 [43,44,45]. Hydrogen atoms were located via a difference Fourier map and refined anistropically. The crystallographic data and structural refinements for complexes **1**–**4** are listed in Table 1. CCDC 1,954,321–1,954,324 contains the supplementary crystallographic data for this paper. These data can be obtained free of charge via http://www.ccdc.cam.ac.uk/conts/retrieving.html (or from the CCDC, 12 Union Road, Cambridge CB2 1EZ, UK; Fax: +44-1223-336033; e-mail: deposit@ccdc.cam.ac.uk).

### 3.7. Peroxidative Reaction of Cyclohexane

In order, we added 3.00 mL acetonitrile, 10.00 mmol H_2_O_2_ (30% in H_2_O), HNO_3_ (0.25 mmol), and 0.20 mL (1.85 mmol) of cyclohexane to the copper catalyst (0.025 mmol) in a reaction flask. The reaction mixture was stirred for 8 h at room temperature in the air under a normal pressure and then treated with excess triphenylphosphine (PPh_3_). This treatment is necessary to neutralize excess hydrogen peroxide and to liberate the cyclohexanol/cyclohexanone products [46,47,48]. After the reaction, 0.20 mL reaction mixture was dissolved in 0.30 mL CDCl_3_ and quantified with the ^1^H NMR technique. The peak (δ = 1.429 ppm, 12H) of cyclohexane, the peaks (δ = 3.578 ppm, 1H) of cyclohexanol, and the peak (δ = 2.335 ppm, 4H) of cyclohexanone were selected as the integrals. The ratios of these peak areas were converted to the ratio of concentration. The yields of cyclohexanol and cyclohexanone could then be calculated. The ^1^H NMR spectrum of the reaction mixture in cyclohexane oxidation catalysed by **4** and an example of the yield calculations are presented in Figure S10.

### 3.8. Gas adsorptions of [Cu_2_(ida)_2_(2-mim)_2_]_n_·nH_2_O (**4**)

Next, **4** (0.20 g) was loaded into a magnetic suspension gravimetric sorption analyser and heated at 423 K under a vacuum to remove water molecules. The temperature was reduced to 298 K until the mass of sample no longer changed. The gas adsorption data were measured by the change in weight under different pressures of O_2_ (CO_2_/CH_4_/N_2_/H_2_) inlet conditions at 298 K.

## 4. Conclusions

We synthesized four 2-methylimidazole copper(II) iminodiacetates [Cu(ida)(2-mim)(H_2_O)_2_]·H_2_O (**1**), [Cu(ida)(2-mim)_2_]·2H_2_O (**2**), [Cu(ida)(2-mim)(H_2_O)]_n_·4.5nH_2_O (**3**), and [Cu_2_(ida)_2_(2-mim)_2_]_n_·nH_2_O (**4**) under different pH values and temperature. We observed holes of ~2.9 Å in diameter in **4** that can adsorb O_2_ selectively and reversibly and remain stable up to 150 °C. The copper complexes show the catalytic activity of the conversions of cyclohexane to cyclohexanol and cyclohexanone, with yields of 18–31% under mild conditions.

## Figures and Tables

**Table 1:** Crystallographic data and structural refinements for the complexes [Cu(ida)(2-mim)(H_2_O)_2_]·H_2_O (**1**), [Cu(ida)(2-mim)_2_]·2H_2_O (**2**), [Cu(ida)(2-mim)(H_2_O)]_n_·4.5nH_2_O (**3**), and [Cu_2_(ida)_2_(2-mim)_2_]_n_·nH_2_O (**4**).

	1	2	3	4
Empirical formula	C_8_H_17_CuN_3_O_7_	C_12_H_21_CuN_5_O_6_	C_18_H_22_CuN_3_O_9.5_	C_16_H_24_Cu_2_N_6_O_9_
Formula weight	330.78	394.88	375.82	571.49
Temperature/K	193	193	193	193
Crystal system	triclinic	triclinic	monoclinic	monoclinic
Space group	*P* ī	*P* ī	*P* 2*_1_*/n	*P* 2*_1_*/n
a/Å	6.9077(8)	7.9289(6)	19.2213(9)	7.7688(8)
b/Å	7.4085(9)	9.4080(8)	7.0465(2)	7.5427(6)
c/Å	13.596(2)	13.1521(9)	24.9007(11)	36.623(2)
α/°	82.241(10)	96.290(6)	90	90
β/°	80.866(10)	105.071(6)	111.120(5)	93.017(7)
γ/°	69.498(11)	111.003(7)	90	90
Volume/Å^3^	641.09(14)	861.74(12)	3146.1(2)	2143.1(3)
Z	2	2	8	4
ρ_calc_ g/cm^3^	1.714	1.522	1.587	1.771
μ/mm^−1^	1.737	1.305	1.438	2.047
F(000)	342	410	1568	1168
Crystal size/mm^3^	0.2 × 0.1× 0.1	0.3 × 0.3 × 0.1	0.3 × 0.3 × 0.1	0.2 × 0.2 × 0.04
Radiation	MoKα (λ = 0.71073)			
*2*θ range for data collection/°	5.892 to 58.174	5.684 to 57.736	4.544 to 60.022	5.31 to 59.998
Reflections collected	4423	6142	16265	9711
*Rint*	0.0269	0.0325	0.0322	0.0582
Data/restraints/parameters	2890/0/184	3874/0/225	8023/0/423	5400/0/303
Goodness of fit on F^2^	1.041	1.052	1.054	1.055
Final *R* indexes [*I ≥ 2σ (I)*]	*R*_1_ = 0.0331 *wR*_2_ = 0.0740	*R*_1_ = 0.0356 *wR*_2_ = 0.0794	*R*_1_ = 0.0399 *wR*_2_ = 0.0948	*R*_1_ = 0.0603 *wR*_2_ = 0.1081
Final *R* indexes [all data]	*R*_1_ = 0.0379 *wR*_2_ = 0.0776	*R*_1_ = 0.0406 *wR*_2_ = 0.0821	*R*_1_ = 0.0558 *wR*_2_ = 0.1051	*R*_1_ = 0.0968 *wR*_2_ = 0.1255
Largest difference peak/hole/e Å^−3^	0.38/−0.56	0.40/−0.62	0.52/−0.90	0.60/−0.77

**Table 2:** Peroxidative reactions of cyclohexane catalysed by complexes **1**–**4** and CuCl_2_^a^.

Catalysts	Yields (%) ^b^		
	Cyclohexanol	Cyclohexanone	Total ^c^
**1**	16	6	22
**2**	13	5	18
**3**	17	7	24
**4**	21	10	31
CuCl_2_	5	5	10
—	0	0	0

^a^ Reaction conditions: catalytic precursor (0.025 mmol), H_2_O_2_ (10.00 mmol), HNO3 (0.25 mmol), C_6_H_12_ (1.85 mmol), excess triphenylphosphine, reaction time 8 h, and room temperature. ^b^ (Moles of product/moles of cyclohexane) × 100%. ^c^ Cyclohexanol + cyclohexanone.

## Supplementary Materials

The following are available online at https://www.mdpi.com/1420-3049/25/6/1286/s1, IR spectra, TG-DTG analyses, ^1^H NMR spectrum, selected bond lengths (Å) and angles (°), hydrogen bonding and packing diagrams of crystal structures, bond valence calculations and detailed adsorption data of O_2_, N_2_, H_2_, CO_2,_ and CH_4_.

## References

[1] Tapiero H., Townsend D.M., Tew K.D. (2003). Trace elements in human physiology and pathology. Copper. Biomed. Pharmacother..

[2] Khan M.H., Cai M., Deng J., Yu P., Liang H., Yang F. (2019). Anticancer function and ros-mediated multi-targeting anticancer mechanisms of copper (II) 2-hydroxy-1-naphthaldehyde complexes. Molecules.

[3] Khanfar M.A., Jaber A.M., Aldamen M.A., Al-Qawasmeh R.A. (2018). Synthesis, characterization, crystal structure, and DFT study of a new square planar Cu(II) complex containing bulky adamantane ligand. Molecules.

[4] Pahon u.E., Paraschivescu C.a., Ilie D.-C., Poirier D., Oprean C., Paunescu V., Gulea A., Ro u.T., Bratu O. (2016). Synthesis and characterization of novel Cu(II), Pd(II) and Pt(II) complexes with 8-ethyl-2-hydroxytricyclo(7.3.1.0(2,7))tridecan-13-one-thiosemicarbazone: Antimicrobial and in vitro antiproliferative activity. Molecules.

[5] Kuckova L., Valko M., Kozisek J., Jomova K., Svorcova A., Seg a.P., Monco J. (2015). Synthesis, crystal structure, spectroscopic properties and potential biological activities of salicylate-neocuproine ternary copper(II) complexes. Molecules.

[6] Ejidike I.P., Ajibade P.A. (2015). Synthesis, characterization and biological studies of metal(II) complexes of (3*E*)-3-[(2-{(*E*)-[1-(2,4-dihydroxyphenyl)ethylidene]amino}ethyl)imino]-1-phenylbutan-1-one Schiff base. Molecules.

[7] Liu J., Chen G., Tan Z. (2016). Copper-catalyzed or -mediated C–H bond functionalizations assisted by bidentate directing groups. Adv. Synth. Catal..

[8] Guo X.X., Gu D.W., Wu Z.X., Zhang W.B. (2015). Copper-catalyzed C–H functionalization reactions: Efficient synthesis of heterocycles. Chem. Rev..

[9] Allen S.E., Walvoord R.R., Padilla-Salinas R., Kozlowski M.C. (2013). Aerobic copper-catalyzed organic reactions. Chem. Rev..

[10] Sen A. (1998). Catalytic functionalization of carbon−hydrogen and carbon−carbon bonds in protic media. Acc. Chem. Res..

[11] Solomon E.I., Heppner D.E., Johnston E.M., Ginsbach J.W., Cirera J., Qayyum M., Kieber-Emmons M.T., Kjaergaard C.H., Hadt R.G., Tian L. (2014). Copper active sites in biology. Chem. Rev..

[12] Gamez P., Aubel P.G., Driessen W.L., Reedijk J. (2001). Homogeneous bio-inspired copper-catalyzed oxidation reactions. Chem. Soc. Rev..

[13] Kirillova M.V., Fernandes T.A., Andre V., Kirillov A.M. (2019). Mild C-H functionalization of alkanes catalyzed by bioinspired copper(II) cores. Org. Biomol. Chem..

[14] Fernandes T.A., André V., Kirillov A.M., Kirillova M.V. (2017). Mild homogeneous oxidation and hydrocarboxylation of cycloalkanes catalyzed by novel dicopper(II) aminoalcohol-driven cores. J. Mol. Catal. A Chem..

[15] Fernandes T.A., Santos C.I.M., André V., Kłak J., Kirillova M.V., Kirillov A.M. (2016). Copper(II) coordination polymers self-assembled from aminoalcohols and pyromellitic acid: Highly active precatalysts for the mild water-promoted oxidation of alkanes. Inorg. Chem..

[16] Chen M.L., Zhou Z.H. (2014). Syntheses and catalytic oxidation of tetrameric and polymeric copper(II) 1,3-propanediaminetetraacetates. Polyhedron.

[17] Di Nicola C., Karabach Y.Y., Kirillov A.M., Monari M., Pandolfo L., Pettinari C., Pombeiro A.J.L. (2007). Supramolecular assemblies of trinuclear triangular copper(II) secondary building units through hydrogen bonds. Generation of different metal−organic frameworks, valuable catalysts for peroxidative oxidation of alkanes. Inorg. Chem..

[18] Kirillov A.M., Kopylovich M.N., Kirillova M.V., Karabach E.Y., Haukka M., da Silva M.F.C.G., Pombeiro A.J.L. (2006). Mild peroxidative oxidation of cyclohexane catalyzed by mono-, di-, tri-, tetra- and polynuclear copper triethanolamine complexes. Adv. Synth. Catal..

[19] Kirillov A.M., Kopylovich M.N., Kirillova M.V., Haukka M., da Silva M.F., Pombeiro A.J. (2005). Multinuclear copper triethanolamine complexes as selective catalysts for the peroxidative oxidation of alkanes under mild conditions. Angew. Chem. Int. Ed. Engl..

[20] Chen M.L., Zhou Z.H. (2017). Structural diversity of 1,3-propylenediaminetetraacetato metal complexes: From coordination monomers to coordination polymers and MOF materials. Inorg. Chim. Acta.

[21] Sakagami N., Yamada Y., Konno T., Okamoto K.-i. (1999). Crystal structures and stereochemical properties of lanthanide(III) complexes with ethylenediamine-*N,N,N′,N′*-tetraacetate. Inorg. Chim. Acta.

[22] Happ B., Winter A., Hager M.D., Schubert U.S. (2012). Photogenerated avenues in macromolecules containing Re(I), Ru(II), Os(II), and Ir(III) metal complexes of pyridine-based ligands. Chem. Soc. Rev..

[23] Hasted J.B. (1969). The structure and properties of water. Phys. Bull..

[24] Speedy R.J., Madura J.D., Jorgensen W.L. (1987). Network topology in simulated water. J. Phys. Chem..

[25] Stupp S.I., Braun P.V. (1997). Molecular manipulation of microstructures: Biomaterials, ceramics, and semiconductors. Science.

[26] Fernandes R.R., Kirillov A.M., Guedes da Silva M.F.C., Ma Z., da Silva J.A.L., Frausto da Silva J.J.R., Pombeiro A.J.L. (2008). An Infinite two-dimensional hybrid water-chloride network, self-assembled in a hydrophobic terpyridine iron(II) matrix. Cryst. Growth Des..

[27] Kirillova M.V., Kirillov A.M., Guedes da Silva M.F.C., Kopylovich M.N., Frausto da Silva J.J.R., Pombeiro A.J.L. (2008). Three-dimensional hydrogen bonded metal-organic frameworks constructed from [M(H_2_O)_6_][M’(dipicolinate)_2_]·mH_2_O (M/M’ = Zn/Ni or Ni/Ni). Identification of intercalated acyclic (H_2_O)_6_/(H_2_O)_10_ clusters. Inorg. Chim. Acta.

[28] Kopylovich M.N., Tronova E.A., Haukka M., Kirillov A.M., Kukushkin V.Y., Frausto da Silva J.J.R., Pombeiro A.J.L. (2007). Identification of hexameric water and hybrid water-chloride clusters intercalated in the crystal hosts of (imidoylamidine)nickel(II) complexes. Eur. J. Inorg. Chem..

[29] Roman-Alpiste M.J., Martin-Ramos J.D., Castineiras-Campos A., Bugella-Altamirano E., Sicilia-Zafra A.G., Gonzalez-Perez J.M., Niclos-Gutierrez J. (1999). Synthesis, XRD structures and properties of diaqua(iminodiacetato)copper(II), [Cu(ida)(H_2_O)_2_], and aqua(benzimidazole)(iminodiacetato)copper(II), [Cu(ida)(HBzIm)(H_2_O)]. Polyhedron.

[30] Castineiras A., Tercero J.M., Matilla A., Gonzalez J.M., Silicia A.G., Niclos J. (1995). Structures and properties of copper(II) complexes with iminodiacetato and imidazole or related ligands. I. Crystal structure of aqua(imidazole)(iminodiacetato)copper(II) monohydrate and (imidazole)(N-carboxymethyl-*D,L*-threoninato)copper(II). J. Coord. Chem..

[31] Castineiras Campos A., Busnot A., Abarca Garcia M.E., Sicilia Zafra A.G., Gonzalez Perez J.M., Niclos Gutierrez J. (1994). Mixed-ligand copper(II) complexes with iminodiacetato and 2- or 4-methylimidazole: Molecular and crystal structure of the ‘remote’ isomer iminodiacetato(5-methylimidazole)copper(II) monohydrate, [Cu(ida)(5MeImH)]·H_2_O. Inorg. Chim. Acta.

[32] Castineiras Campos A., Sicilia Zafra A.G., Gonzalez Perez J.M., Niclos Gutierrez J., Chinea E., Mederos A. (1996). Mixed-ligand copper(II) complexes with N-methyl derivatives of iminodiacetato or imidazole: Crystal structures of (N-methyl-iminodiacetato)(imidazole)copper(II), [Cu(mida)(ImH)] and diaqua(iminodiacetato)(N-methyl-imidazole)copper(II) monohydrate, [Cu(ida)(1MeImH)(H_2_O)_2_]·H_2_O. Inorg. Chim. Acta.

[33] Zhang W.J., Li Y.T., Wu Z.Y., Liu Z.Q., Zheng Z.C. (2008). Synthesis, crystal structure and DNA-binding study of a new copper(II) complex containing mixed-ligand of iminodiacetate dianion and 6-nitro-1H-benzimidazole. J. Chem. Crystallogr..

[34] Craven E., Zhang C., Janiak C., Rheinwald G., Lang H. (2003). Synthesis, structure and solution chemistry of (5,5’-dimethyl-2,2’-bipyridine)(ida)copper(II) and structural comparison with aqua(ida)(1,10-phenanthroline)copper(II) (ida = iminodiacetato). Z. Anorg. Allg. Chem..

[35] Brown I.D., Altermatt D. (1985). Bond-valence parameters obtained from a systematic analysis of the Inorganic Crystal Structure Database. Acta Crystallogr. B.

[36] Zhao N., Li Y., Gu J., Fernandes T.A., Kirillova M.V., Kirillov A.M. (2019). New copper(II) coordination compounds assembled from multifunctional pyridine-carboxylate blocks: Synthesis, structures, and catalytic activity in cycloalkane oxidation. Molecules.

[37] Dias S.S.P., Kirillova M.V., André V., Kłak J., Kirillov A.M. (2015). New tetracopper(II) cubane cores driven by a diamino alcohol: Self-assembly Synthesiss, structural and topological features, and magnetic and catalytic oxidation properties. Inorg. Chem..

[38] Czerwinska K., Machura B., Kula S., Krompiec S., Erfurt K., Roma-Rodrigues C., Fernandes A.R., Shul’pina L.S., Ikonnikov N.S., Shul’pin G.B. (2017). Copper(II) complexes of functionalized 2,2’:6’,2’’-terpyridines and 2,6-di(thiazol-2-yl)pyridine: Structure, spectroscopy, cytotoxicity and catalytic activity. Dalton Trans..

[39] Garcia-Bosch I., Siegler M.A. (2016). Copper-catalyzed oxidation of alkanes with H_2_O_2_ under a fenton-like regime. Angew. Chem. Int. Ed..

[40] Kirillova M.V., Santos C.I.M., Wu W., Tang Y., Kirillov A.M. (2017). Mild oxidative C-H functionalization of alkanes and alcohols using a magnetic core-shell Fe_3_O_4_@mSiO_2_@Cu_4_ nanocatalyst. J. Mol. Catal. A Chem..

[41] Dias S.S.P., Kirillova M.V., Andre V., Klak J., Kirillov A.M. (2015). New tricopper(II) cores self-assembled from aminoalcohol biobuffers and homophthalic acid: Synthesis, structural and topological features, magnetic properties and mild catalytic oxidation of cyclic and linear C5-C8 alkanes. Inorg. Chem. Front..

[42] Farrugia L.J. (2012). WinGX and ORTEP for Windows: An update. J. Appl. Crystallogr..

[43] Sheldrick G. (2015). Crystal structure refinement with SHELXL. Acta Crystallogr. C.

[44] Dolomanov O.V., Bourhis L.J., Gildea R.J., Howard J.A.K., Puschmann H. (2009). OLEX2: A complete structure solution, refinement and analysis program. J. Appl. Crystallogr..

[45] Sheldrick G. (2008). A short history of SHELX. Acta Crystallogr. A.

[46] Shul’pin G.B. (2009). Hydrocarbon oxygenations with peroxides catalyzed by metal compounds. Mini-Rev. Org. Chem..

[47] Shul’pin G.B. (2003). Metal-catalysed hydrocarbon oxidations. Comptes Rendus Chimie.

[48] Shul’pin G.B. (2002). Metal-catalyzed hydrocarbon oxygenations in solutions: The dramatic role of additives: A review. J. Mol. Catal. A Chem..

