# Supplementary material for: 2-Methylimidazole Copper Iminodiacetates for the Adsorption of Oxygen and Catalytic Oxidation of Cyclohexane
Source: Molecules. 2020 Mar 12;25(6):1286. doi: 10.3390/molecules25061286 (PMC7143979; doi:10.3390/molecules25061286)

checkCIF/PLATON report

Structure factors have been supplied for datablock(s) exp_21035

THIS REPORT IS FOR GUIDANCE ONLY. IF USED AS PART OF A REVIEW PROCEDURE FOR PUBLICATION, IT SHOULD NOT REPLACE THE EXPERTISE OF AN EXPERIENCED CRYSTALLOGRAPHIC REFEREE.

No syntax errors found.   CIF dictionary   Interpreting this report

Datablock: exp_21035

```
Bond precision:  C-C = 0.0040 A          Wavelength=0.71073

Cell:           a=7.9289(6)       b=9.4080(8)       c=13.1521(9)
                alpha=96.290(6)   beta=105.071(6)   gamma=111.003(7)
Temperature:    193 K

                Calculated                 Reported
Volume          861.74(13)                 861.74(12)
Space group     P -1                       P -1
Hall group      -P 1                       -P 1
Moiety formula  C12 H17 Cu N5 O4, 2(H2 O)  C12 H17 Cu N5 O4, 2(H2 O)
Sum formula     C12 H21 Cu N5 O6           C12 H21 Cu N5 O6
Mr              394.89                     394.88
Dx,g cm-3       1.522                      1.522
Z               2                          2
Mu (mm-1)       1.305                      1.305
F000            410.0                      410.0
F000'           410.81
h,k,lmax        10,12,17                   10,12,17
Nref            4533                       3874
Tmin,Tmax       0.683,0.878                0.795,1.000
Tmin'           0.669

Correction method= # Reported T Limits: Tmin=0.795 Tmax=1.000
AbsCorr = MULTI-SCAN

Data completeness= 0.855          Theta(max)= 28.868

R(reflections)= 0.0356( 3540)     wR2(reflections)= 0.0821( 3874)

S = 1.052                   Npar= 225
```

The following ALERTS were generated. Each ALERT has the format
test-name_ALERT_alert-type_alert-level.
Click on the hyperlinks for more details of the test.

Alert level C

```
PLAT411_ALERT_2_C Short Inter H...H Contact  H2A      ..H2A      .       2.05 Ang.
                                          1-x,-y,1-z  =      2_656 Check
PLAT413_ALERT_2_C Short Inter XH3 .. XHn     H6A      ..H10A     .       2.12 Ang.
                                            -1+x,y,z  =      1_455 Check
PLAT977_ALERT_2_C Check Negative Difference Density on H6A              -0.36 eA-3
```

Alert level G

```
PLAT007_ALERT_5_G Number of Unrefined Donor-H Atoms .............          7 Report
PLAT720_ALERT_4_G Number of Unusual/Non-Standard Labels ..........          4 Note
PLAT794_ALERT_5_G Tentative Bond Valency for Cu1       (II)      .       2.17 Info
PLAT910_ALERT_3_G Missing # of FCF Reflection(s) Below Theta(Min).          3 Note
PLAT912_ALERT_4_G Missing # of FCF Reflections Above STh/L=  0.600        656 Note
PLAT933_ALERT_2_G Number of OMIT Records in Embedded .res File ...          2 Note
PLAT978_ALERT_2_G Number C-C Bonds with Positive Residual Density.          1 Info
PLAT992_ALERT_5_G Repd & Actual _reflns_number_gt Values Differ by          1 Check
```

```
   0 ALERT level A = Most likely a serious problem - resolve or explain
   0 ALERT level B = A potentially serious problem, consider carefully
   3 ALERT level C = Check. Ensure it is not caused by an omission or oversight
   8 ALERT level G = General information/check it is not something unexpected

   0 ALERT type 1 CIF construction/syntax error, inconsistent or missing data
   5 ALERT type 2 Indicator that the structure model may be wrong or deficient
   1 ALERT type 3 Indicator that the structure quality may be low
   2 ALERT type 4 Improvement, methodology, query or suggestion
   3 ALERT type 5 Informative message, check
```

It is advisable to attempt to resolve as many as possible of the alerts in all categories. Often the minor alerts point to easily fixed oversights, errors and omissions in your CIF or refinement strategy, so attention to these fine details can be worthwhile. In order to resolve some of the more serious problems it may be necessary to carry out additional measurements or structure refinements. However, the purpose of your study may justify the reported deviations and the more serious of these should normally be commented upon in the discussion or experimental section of a paper or in the "special_details" fields of the CIF. checkCIF was carefully designed to identify outliers and unusual parameters, but every test has its limitations and alerts that are not important in a particular case may appear. Conversely, the absence of alerts does not guarantee there are no aspects of the results needing attention. It is up to the individual to critically assess their own results and, if necessary, seek expert advice.

Publication of your CIF in IUCr journals

A basic structural check has been run on your CIF. These basic checks will be run on all CIFs submitted for publication in IUCr journals (*Acta Crystallographica*, *Journal of Applied Crystallography*, *Journal of Synchrotron Radiation*); however, if you intend to submit to *Acta Crystallographica Section C* or *E* or *IUCrData*, you should make sure that full publication checks are run on the final version of your CIF prior to submission.

Publication of your CIF in other journals

Please refer to the *Notes for Authors* of the relevant journal for any special instructions relating to CIF submission.

PLATON version of 07/08/2019; check.def file version of 30/07/2019

Datablock exp_21035 - ellipsoid plot

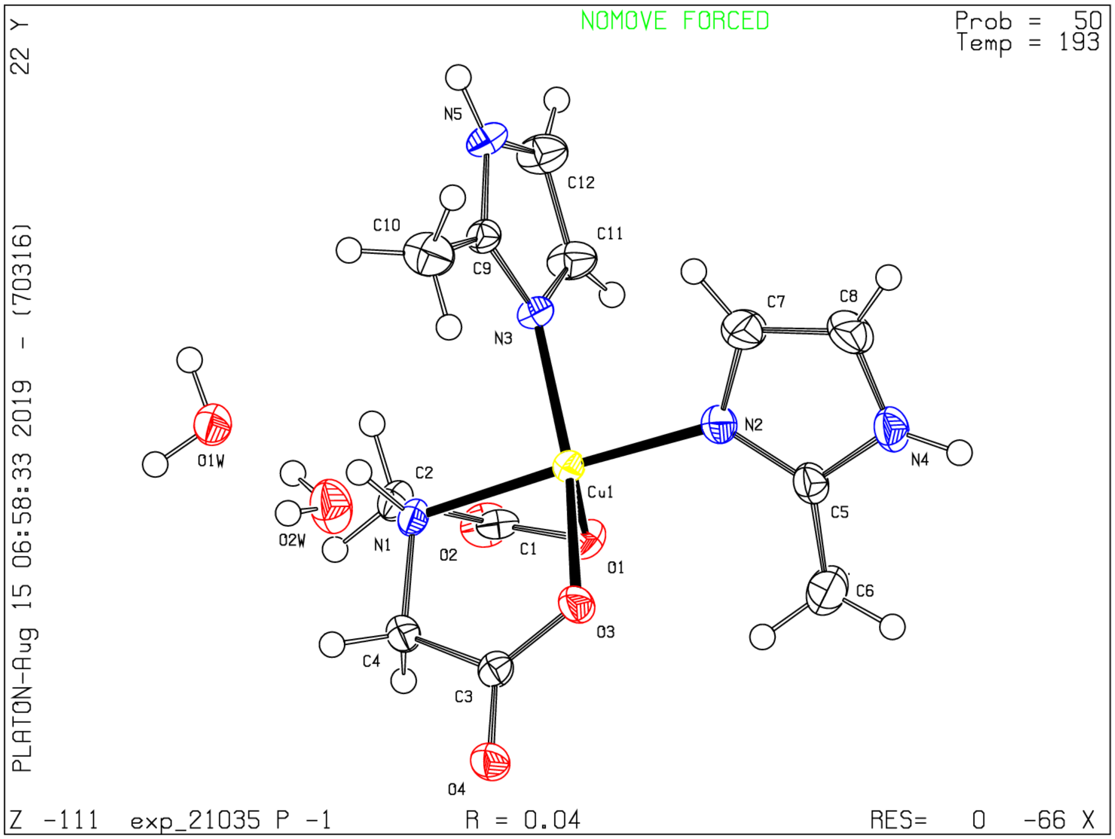

Supplement: Supplementary file 1 [file molecules-25-01286-s001.zip › Supplementary Files/Checkcif2.pdf]
