# Supplementary material for: 2-Methylimidazole Copper Iminodiacetates for the Adsorption of Oxygen and Catalytic Oxidation of Cyclohexane
Source: Molecules. 2020 Mar 12;25(6):1286. doi: 10.3390/molecules25061286 (PMC7143979; doi:10.3390/molecules25061286)

checkCIF/PLATON report

Structure factors have been supplied for datablock(s) exp_20419

THIS REPORT IS FOR GUIDANCE ONLY. IF USED AS PART OF A REVIEW PROCEDURE FOR PUBLICATION, IT SHOULD NOT REPLACE THE EXPERTISE OF AN EXPERIENCED CRYSTALLOGRAPHIC REFEREE.

No syntax errors found. CIF dictionary Interpreting this report

Datablock: exp_20419

```
Bond precision:  C-C = 0.0030 A          Wavelength=0.71073

Cell:           a=19.2213(9)    b=7.0465(2)         c=24.9007(11)
                alpha=90        beta=111.120(5)     gamma=90
Temperature:    193 K

                Calculated                         Reported
Volume          3146.1(2)                          3146.1(2)
Space group     P 21/n                             P 1 21/n 1
Hall group      -P 2yn                             -P 2yn
Moiety formula  2(C8 H13 Cu N3 O5), 9(H2 O)        C8 H11 Cu N3 O4, 5.5(H2 O)
Sum formula     C16 H44 Cu2 N6 O19                 C8 H22 Cu N3 O9.50
Mr              751.67                             375.82
Dx,g cm-3       1.587                              1.587
Z               4                                  8
Mu (mm-1)       1.438                              1.438
F000            1568.0                             1568.0
F000'           1571.41
h,k,lmax        27,9,35                            26,9,34
Nref            9167                               8023
Tmin,Tmax       0.656,0.866                        0.713,1.000
Tmin'           0.643

Correction method= # Reported T Limits: Tmin=0.713 Tmax=1.000
AbsCorr = MULTI-SCAN

Data completeness= 0.875          Theta(max)= 30.011

R(reflections)= 0.0399( 6271)     wR2(reflections)= 0.1052( 8023)

S = 1.054                  Npar= 423
```

The following ALERTS were generated. Each ALERT has the format
test-name_ALERT_alert-type_alert-level.
Click on the hyperlinks for more details of the test.

Alert level C

```
PLAT112_ALERT_2_C ADDSYM Detects New (Pseudo) Symm. Elem         A         88 %Fit
PLAT906_ALERT_3_C Large K Value in the Analysis of Variance ......      3.354 Check
PLAT934_ALERT_3_C Number of (Iobs-Icalc)/Sigma(W) > 10 Outliers ..          1 Check
PLAT978_ALERT_2_C Number C-C Bonds with Positive Residual Density.          0 Info
```

Alert level G

```
PLAT004_ALERT_5_G Polymeric Structure Found with Maximum Dimension          1 Info
PLAT007_ALERT_5_G Number of Unrefined Donor-H Atoms .............          26 Report
PLAT042_ALERT_1_G Calc. and Reported MoietyFormula Strings  Differ     Please Check
PLAT045_ALERT_1_G Calculated and Reported Z Differ by a Factor ...       0.50 Check
PLAT232_ALERT_2_G Hirshfeld Test Diff (M-X)  Cu1      --N1       .        6.1 s.u.
PLAT232_ALERT_2_G Hirshfeld Test Diff (M-X)  Cu2      --N4       .        5.4 s.u.
PLAT720_ALERT_4_G Number of Unusual/Non-Standard Labels ..........         18 Note
PLAT794_ALERT_5_G Tentative Bond Valency for Cu1       (II)      .       2.18 Info
PLAT794_ALERT_5_G Tentative Bond Valency for Cu2       (II)      .       2.18 Info
PLAT910_ALERT_3_G Missing # of FCF Reflection(s) Below Theta(Min).          3 Note
PLAT912_ALERT_4_G Missing # of FCF Reflections Above STh/L=  0.600       1136 Note
PLAT913_ALERT_3_G Missing # of Very Strong Reflections in FCF ....          1 Note
```

---

0 **ALERT level A** = Most likely a serious problem - resolve or explain
0 **ALERT level B** = A potentially serious problem, consider carefully
4 **ALERT level C** = Check. Ensure it is not caused by an omission or oversight
12 **ALERT level G** = General information/check it is not something unexpected

2 ALERT type 1 CIF construction/syntax error, inconsistent or missing data
4 ALERT type 2 Indicator that the structure model may be wrong or deficient
4 ALERT type 3 Indicator that the structure quality may be low
2 ALERT type 4 Improvement, methodology, query or suggestion
4 ALERT type 5 Informative message, check

It is advisable to attempt to resolve as many as possible of the alerts in all categories. Often the minor alerts point to easily fixed oversights, errors and omissions in your CIF or refinement strategy, so attention to these fine details can be worthwhile. In order to resolve some of the more serious problems it may be necessary to carry out additional measurements or structure refinements. However, the purpose of your study may justify the reported deviations and the more serious of these should normally be commented upon in the discussion or experimental section of a paper or in the "special_details" fields of the CIF. checkCIF was carefully designed to identify outliers and unusual parameters, but every test has its limitations and alerts that are not important in a particular case may appear. Conversely, the absence of alerts does not guarantee there are no aspects of the results needing attention. It is up to the individual to critically assess their own results and, if necessary, seek expert advice.

Publication of your CIF in IUCr journals

A basic structural check has been run on your CIF. These basic checks will be run on all CIFs submitted for publication in IUCr journals (*Acta Crystallographica*, *Journal of Applied Crystallography*, *Journal of Synchrotron Radiation*); however, if you intend to submit to *Acta Crystallographica Section C* or *E* or *IUCrData*, you should make sure that full publication checks are run on the final version of your CIF prior to submission.

Publication of your CIF in other journals

Please refer to the *Notes for Authors* of the relevant journal for any special instructions relating to CIF submission.

PLATON version of 07/08/2019; check.def file version of 30/07/2019

Datablock exp_20419 - ellipsoid plot

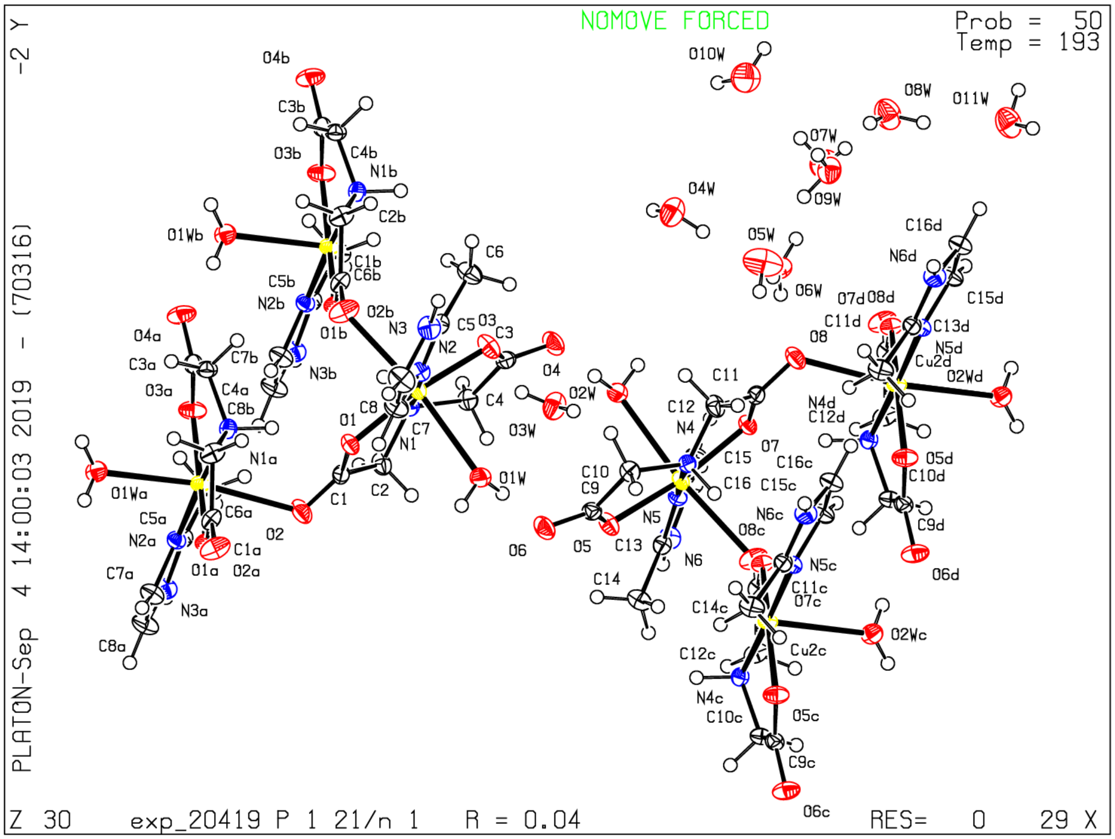

Supplement: Supplementary file 1 [file molecules-25-01286-s001.zip › Supplementary Files/Checkcif3.pdf]
