# Supplementary material for: 2-Methylimidazole Copper Iminodiacetates for the Adsorption of Oxygen and Catalytic Oxidation of Cyclohexane
Source: Molecules. 2020 Mar 12;25(6):1286. doi: 10.3390/molecules25061286 (PMC7143979; doi:10.3390/molecules25061286)
Supplement: Supplementary file 1 [file molecules-25-01286-s001.zip › Supplementary Files/Supporting Information.docx]

Supporting information

**2-Methylimidazole copper iminodiacetates for the adsorption of oxygen and catalytic oxidation of cyclohexane**

Xi Chen ^1^, Dong-Li An ^1^, Xin-Qi Zhan ^2^ and Zhao-Hui Zhou^1,*^

*^1^ State Key Laboratory for Physical Chemistry of Solid Surfaces, College of Chemistry and Chemical Engineering, Xiamen University, Xiamen, 361005, China; xichen@stu.xmu.edu.cn (X.C.); andongli@xmu.edu.cn (D.A.)*

*^2^ Medical College, Xiamen University, Xiamen, 361005, China; zxq@xmu.edu.cn*

** Correspondence: zhzhou@xmu.edu.cn; Tel.: + 86-592-2184531*

**Figures and Table Options**

Figure S1. 2D structure of [Cu(ida)(2-mim)(H_2_O)_2_]·H_2_O (**1**).

Figure S2. 2D structure of [Cu(ida)(2-mim)_2_]·2H_2_O (**2**).

Figure S3. 2D structure of [Cu(ida)(2-mim)(H_2_O)]_n_·4.5nH_2_O (**3**).

Figure S4. 2D structure of [Cu_2_(ida)_2_(2-mim)_2_]_n_·nH_2_O (**4**).

Figure S5. Schematic descriptions of the equivalent topology frameworks in [Cu_2_(ida)_2_(2-mim)_2_]_n_·nH_2_O (**4**). Color codes: sky blue for [Cu_2_(ida)_2_(2-mim)_2_] units.

Figure S6. IR spectra of [Cu(ida)(2-mim)(H_2_O)_2_]·H_2_O (**1**), [Cu(ida)(2-mim)_2_]·2H_2_O (**2**), [Cu(ida)(2-mim)(H_2_O)]_n_·4.5nH_2_O (**3**) and [Cu_2_(ida)_2_(2-mim)_2_]_n_·nH_2_O (**4**).

Figure S7. TG-DTG curves of [Cu(ida)(2-mim)(H_2_O)_2_]·H_2_O (**1**).

Figure S8. TG-DTG curves of [Cu(ida)(2-mim)_2_]·2H_2_O (**2**).

Figure S9. TG-DTG curves of [Cu(ida)(2-mim)(H_2_O)]_n_·4.5nH_2_O (**3**).

Figure S10. ^1^H NMR spectrum of the reaction mixture in cyclohexane oxidation catalysed by **4**.

Table S1. Comparisons of selected bond lengths () for [Cu(ida)(2-mim)(H_2_O)_2_]·H_2_O (**1**), [Cu(ida)(2-mim)_2_]·2H_2_O (**2**), [Cu(ida)(2-mim)(H_2_O)]_n_·4.5nH_2_O (**3**), [Cu_2_(ida)_2_(2-mim)_2_]_n_·nH_2_O (**4**) and the other copper(II) iminodiacetates.

Table S2. Hydrogen bond lengths () and angles (°) in [Cu(ida)(2-mim)(H_2_O)_2_]·H_2_O (**1**).

Table S3. Hydrogen bond lengths () and angles (°) in [Cu(ida)(2-mim)_2_]·2H_2_O (**2**).

Table S4. Hydrogen bond lengths () and angles (°) in [Cu(ida)(2-mim)(H_2_O)]_n_·4.5nH_2_O (**3**).

Table S5. Hydrogen bond lengths () and angles (°) in [Cu_2_(ida)_2_(2-mim)_2_]_n_·nH_2_O (**4**).

Table S6. Selected bond lengths (Å) and angles (º) for [Cu(ida)(2-mim)(H_2_O)_2_]·H_2_O (**1**).

Table S7. Selected bond lengths (Å) and angles (º) for [Cu(ida)(2-mim)_2_]·2H_2_O (**2**).

Table S8. Selected bond lengths (Å) and angles (º) for [Cu(ida)(2-mim)(H_2_O)]_n_·4.5nH_2_O (**3**).

Table S9. Selected bond lengths (Å) and angles (º) for [Cu_2_(ida)_2_(2-mim)_2_]_n_·nH_2_O (**4**).

Table S10. Bond valence calculations for **1** ~ **4**.

Table S11. Detailed adsorption data of **4** for O_2_, N_2_, H_2_, CO_2_ and CH_4_ and desorption data for O_2_.

Figure S1. 2D structure of [Cu(ida)(2-mim)(H_2_O)_2_]·H_2_O (**1**).


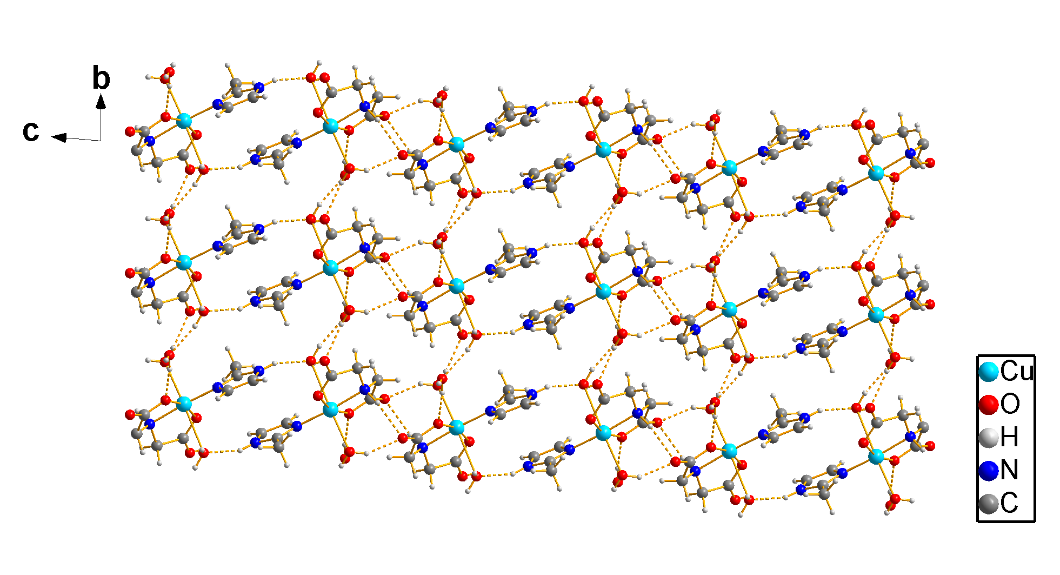


Figure S2. 2D structure of [Cu(ida)(2-mim)_2_]·2H_2_O (**2**).


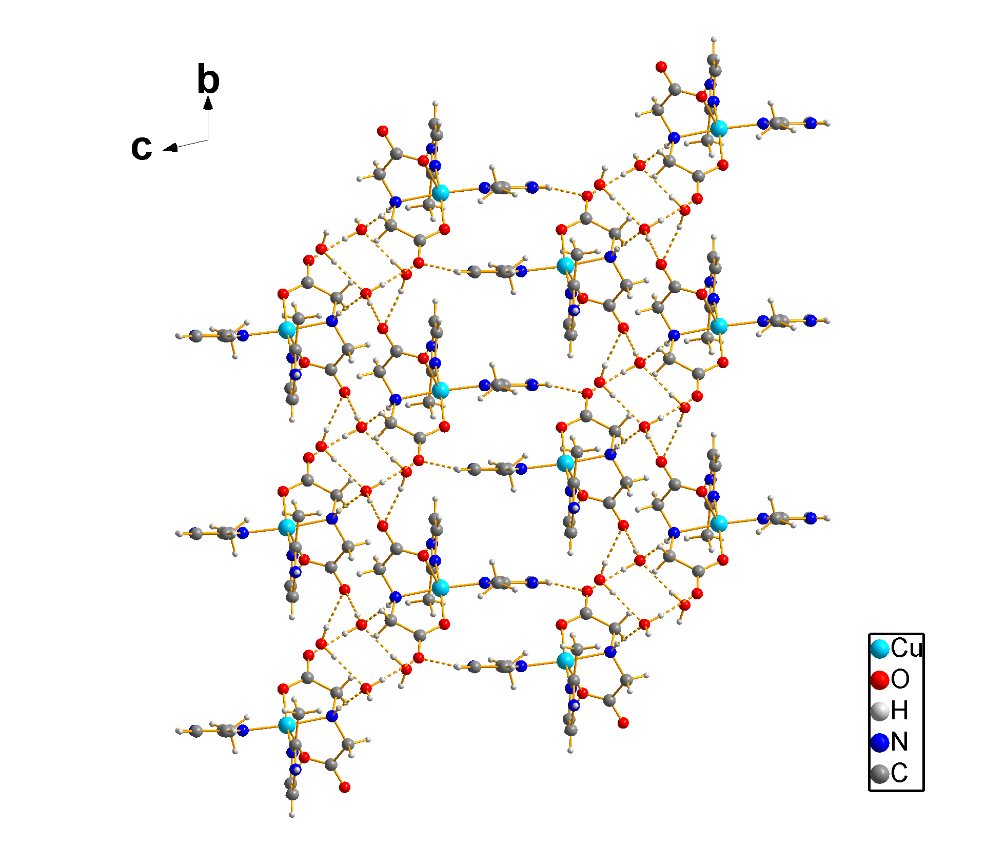


Figure S3. 2D structure of [Cu(ida)(2-mim)_n_]·4.5nH_2_O (**3**).


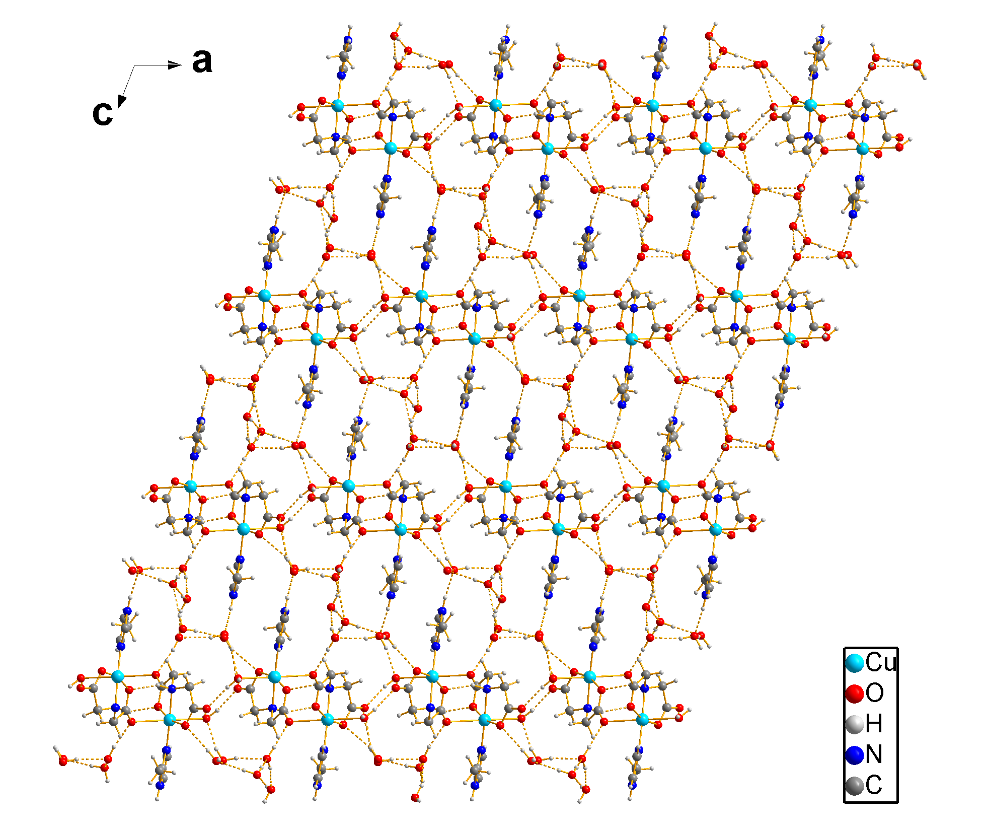


Figure S4. 2D structure of [Cu_2_(ida)_2_(2-mim)_2_]_n_·nH_2_O (**4**).


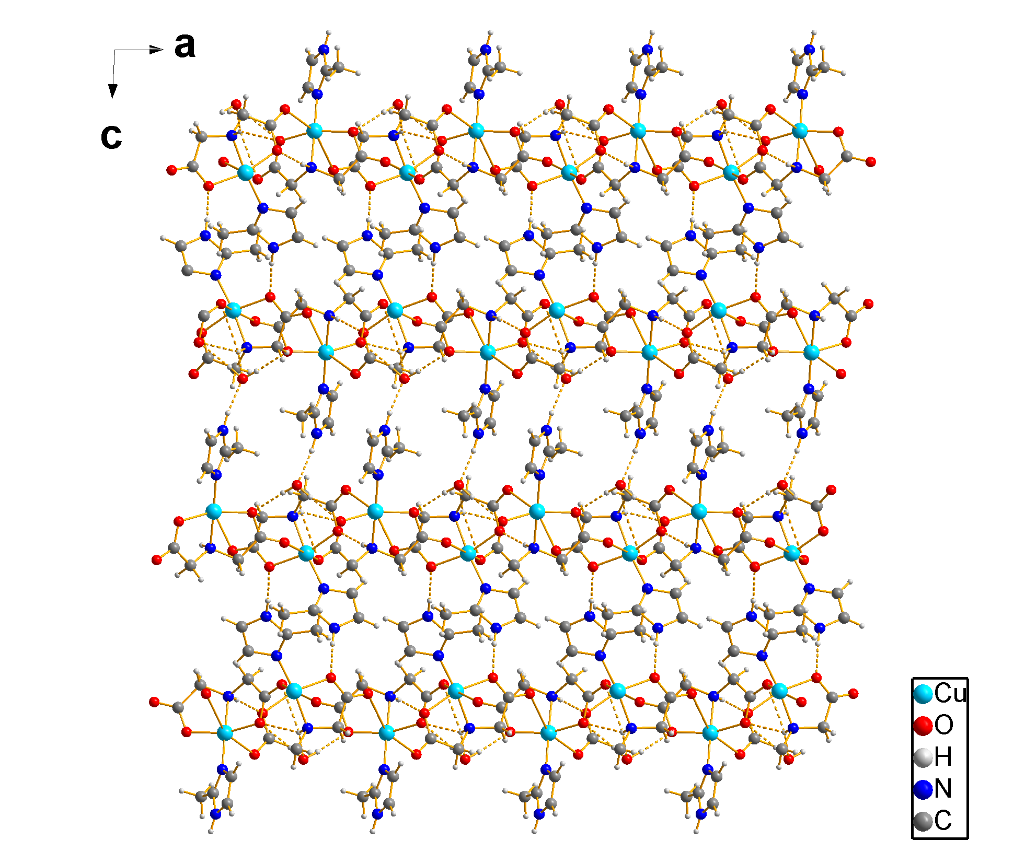


Figure S5. Schematic descriptions of the equivalent topology frameworks in [Cu_2_(ida)_2_(2-mim)_2_]_n_·nH_2_O (**4**). Color codes: sky blue for [Cu_2_(ida)_2_(2-mim)_2_] units.


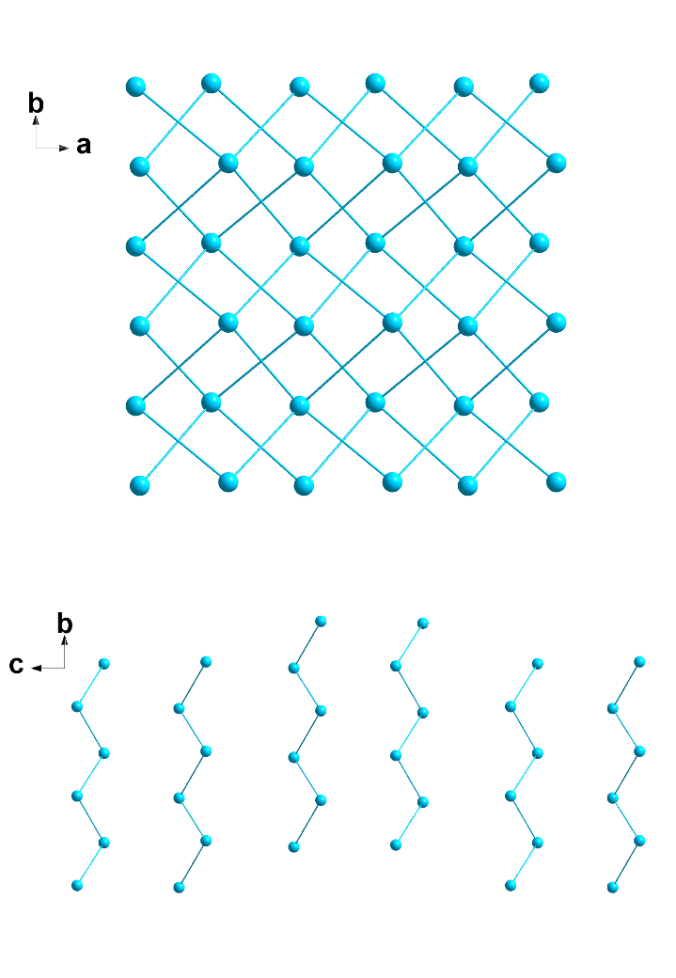


Figure S6. IR spectra of [Cu(ida)(2-mim)(H_2_O)_2_]·H_2_O (**1**), [Cu(ida)(2-mim)_2_]·2H_2_O (**2**), [Cu(ida)(2-mim)(H_2_O)]_n_·4.5nH_2_O (**3**) and [Cu_2_(ida)_2_(2-mim)_2_]_n_·nH_2_O (**4**).





Figure S7. TG-DTG curves of [Cu(ida)(2-mim)(H_2_O)_2_]·H_2_O (**1**).





Figure S8. TG-DTG curves of [Cu(ida)(2-mim)_2_]·2H_2_O (**2**).





Figure S9. TG-DTG curves of [Cu(ida)(2-mim)(H_2_O)]_n_·4.5nH_2_O (**3**).





Figure S10. ^1^H NMR spectrum of the reaction mixture in cyclohexane oxidation catalysed by **4**. σ (ppm): 1.429 (cyclohexane), 2.335 (cyclohexanone), 3.578 (cyclohexanol).





Y_cyclohexanol_(%)=$\frac{A_{\mathrm{cyclohexanol}}}{A_{\mathrm{cyclohexanol}}+\frac{A_{\mathrm{cyclohexanone}}}{4}+\frac{A_{\mathrm{cyclohexane}}}{12}}\times100\%$=$\frac{0.03}{0.03+\frac{0.06}{4}+\frac{1.22}{12}}\times100\%$= 21%

Y_cyclohexanone_(%)=$\frac{\frac{A_{\mathrm{cyclohexanone}}}{4}}{A_{\mathrm{cyclohexanol}}+\frac{A_{\mathrm{cyclohexanone}}}{4}+\frac{A_{\mathrm{cyclohexane}}}{12}}\times100\%$=$\frac{\frac{0.06}{4}}{0.03+\frac{0.06}{4}+\frac{1.22}{12}}\times100\%$= 10%

Y_cyclohexanol_: The yield of cyclohexanol; Y_cyclohexanone_: The yield of cyclohexanone

A_cyclohexanol_: The peak area of cyclohexanol; A_cyclohexanone_: The peak area of cyclohexanone; A_cyclohexane_: The peak area of cyclohexane.

Table S1. Comparisons of selected bond lengths () for [Cu(ida)(2-mim)(H_2_O)_2_]·H_2_O (**1**), [Cu(ida)(2-mim)_2_]·2H_2_O (**2**), [Cu(ida)(2-mim)(H_2_O)]_n_·4.5nH_2_O (**3**), [Cu_2_(ida)_2_(2-mim)_2_]_n_·nH_2_O (**4**) and the other copper(II) iminodiacetates.

| Complexes | Cu−O in plane | Cu−O axial | Cu−N_ida_ | Cu−N_2-mim_ |
| --- | --- | --- | --- | --- |
| [Cu(ida)(2-mim)(H_2_O)_2_]·H_2_O (**1**) | 1.985(2)_av_ | 2.580(2)_av_ | 1.998(2) | 1.955(2) |
| [Cu(ida)(2-mim)_2_]·2H_2_O (**2**) | 1.962 (2) | 2.300(2) | 2.042(2) | 1.985(2)_av_ |
| [Cu(ida)(2-mim)(H_2_O)]_n_·4.5nH_2_O (**3**) | 1.997(2)_av_ | 2.481(2)_av_ | 1.991(2) | 1.953(2) |
| [Cu_2_(ida)_2_(2-mim)_2_]_n_·nH_2_O (**4**) | 1.991(3)_av_ | 2.478(3)_av_ | 1.974(3) | 1.965(3) |
| [Cu(ida)(H_2_O)_2_]_n_ | 1.962(1)_av_ | 2.440(2) | 2.015(1) |  |
| [Cu(ida)(im)(H_2_O)]·H_2_O | 1.99(1)_av_ | 2.37(1) | 2.01(1) | 1.94(2) |
| [Cu(ida)(5-mim)]·H_2_O | 1.97(1)_av_ | 2.41(1) | 1.99(1) | 1.93(1) |
| [Cu(ida)(1-mim)(H_2_O)_2_]·H_2_O | 1.986(5) | 2.511(6) | 1.990(4) | 1.938(4) |
| [Cu(ida)(HBzIm)(H_2_O)] | 1.974(3)_av_ | 2.357(4)_av_ | 1.996(3) | 1.956(3) |
| [Cu(ida)(NBzIm)(H_2_O)] | 1.968(3)_av_ | 2.363(4) | 2.005(3) | 1.960(3) |
| [Cu(ida)(bpy)]·4H_2_O | 1.953(2) | 2.190(3) | 2.042(3) | 2.005(3) |
| [Cu(ida)(H_2_O)(phen)]·4H_2_O | 1.973(2) | 2.373(2) | 2.030(2) | 2.022(2) |

Av: average; im: imidazole; 5-mim: 5-methylimidazole; 1-mim: 1-methylimidazole; HBzIm: benzimidazole; NbzIm: 6-nitro-1H-benzimidazole; bpy: 5,5-dimethyl-2,2-bipyridine; phen: 1,10-phenanthroline.

Table S2. Hydrogen bonds lengths () and angles (°) in [Cu(ida)(2-mim)(H_2_O)_2_]·H_2_O (**1**).

| Donor–H…Acceptor | D–H | H…A | D…A | D–H…A |
| --- | --- | --- | --- | --- |
| N1–H1···O2a | 1.00 | 2.24 | 3.035(3) | 136 |
| O2w–H2wA···O3wb | 0.85 | 1.91 | 2.741(3) | 166 |
| O2w–H2wB···O4c | 0.85 | 2.03 | 2.847(3) | 161 |
| N3–H3···O2wd | 0.88 | 2.03 | 2.855(3) | 157 |
| O1w–H1wA···O4e | 0.85(2) | 1.94(2) | 2.784(3) | 171(3) |
| O1w–H1wB···O2a | 0.85(2) | 1.92(2) | 2.733(3) | 158(3) |
| O3w–H3wA···O1 | 0.85 | 1.90 | 2.743(3) | 173 |
| O3w–H3wB···O1wc | 0.85(2) | 1.94(4) | 2.759(3) | 160(3) |

Symmetry codes: (a) 1 – *x*, 1 – *y*, 2 – *z*; (b) *x,* -1 *+ y*, *z*; (c) 1 + *x*, *y*, *z*; (d) 1 – *x*, 1 – *y*, 1 – *z*; (e) *x*, 1 + *y*, *z*.

Table S3. Hydrogen bonds lengths () and angles (°) in [Cu(ida)(2-mim)_2_]·2H_2_O (**2**).

| Donor–H…Acceptor | D–H | H…A | D…A | D–H…A |
| --- | --- | --- | --- | --- |
| N1–H1···O1w | 1.00 | 2.03 | 2.987(3) | 159 |
| O1w–H1wA···O4a | 0.85 | 1.93 | 2.777(2) | 177 |
| O1w–H1wB···O2b | 0.85 | 2 | 2.846(3) | 173 |
| O2w–H2wA···O2c | 0.85 | 2.01 | 2.855(3) | 172 |
| N4–H4...O4d | 0.88 | 1.96 | 2.824(3) | 168 |
| O2w–H2wB···O1w | 0.85 | 2.02 | 2.853(3) | 167 |
| N5–H5···O1e | 0.88 | 1.93 | 2.780(3) | 162 |

Symmetry codes: (a) 1 – *x*, 1 – *y*, 1 – *z*; (b) 1 – *x*, -*y*, 1 – *z*; (c) 1 + *x*, 1 + *y*, *z*; (d) 1 – *x*, 1 – *y*, 2 – *z*; (e) 1 + *x*, *y*, *z*.

Table S4. Hydrogen bonds lengths () and angles (°) in [Cu(ida)(2-mim)H_2_O]_n_·4.5nH_2_O (**3**).

| Donor–H…Acceptor | D–H | H…A | D…A | D–H…A |
| --- | --- | --- | --- | --- |
| N1–H1···O1a | 1.00 | 1.98 | 2.929(3) | 157 |
| O2w–H2wA···O4 | 0.85 | 1.89 | 2.734(3) | 170 |
| O2w–H2wB···O6b | 0.85 | 1.94 | 2.773(2) | 166 |
| N3–H3···O4wc | 0.88 | 2 | 2.854(3) | 163 |
| O1w–H1wA···O6 | 0.85 | 1.9 | 2.750(3) | 173 |
| N4–H4···O7d | 1.00 | 1.95 | 2.899(3) | 158 |
| O1w–H1wB···O4e | 0.85 | 1.96 | 2.796(2) | 167 |
| O9w–H9wA···O5w | 0.85 | 1.86 | 2.691(3) | 167 |
| N6–H6···O3wf | 0.88 | 1.94 | 2.814(3) | 169 |
| O9w–H9wB···O2g | 0.85 | 1.9 | 2.747(3) | 175 |
| O3w–H3wA···O2w | 0.85 | 1.95 | 2.766(2) | 160 |
| O3w–H3wB···O9wh | 0.85 | 1.94 | 2.781(3) | 169 |
| O6w–H6wA···O7w | 0.85 | 1.89 | 2.730(3) | 167 |
| O6w–H6wB···O8 | 0.85 | 1.9 | 2.745(3) | 171 |
| O10w–H10C···O3b | 0.85 | 2.44 | 3.232(3) | 154 |
| O10w–H10C···O4b | 0.85 | 2.46 | 3.223(3) | 150 |
| O10w–H10D···O4wb | 0.85 | 2.1 | 2.950(3) | 173 |
| O4w–H4wA···O6w | 0.85 | 1.93 | 2.770(3) | 169 |
| O11w–H11A···O5i | 0.85 | 2.21 | 3.052(3) | 169 |
| O11w–H11B···O3wj | 0.85 | 2.07 | 2.916(3) | 173 |
| O4w–H4wB···O1wb | 0.85 | 2.01 | 2.808(2) | 157 |
| O8w–H8wA···O11w | 0.85 | 1.95 | 2.776(3) | 164 |
| O8w–H8wB···O9w | 0.85 | 1.99 | 2.803(3) | 160 |
| O7w–H7wA···O10w | 0.85 | 2.01 | 2.854(3) | 174 |
| O7w–H7wB···O8w | 0.85 | 1.98 | 2.826(3) | 176 |
| O5w–H5wA···O8we | 0.85 | 2.01 | 2.848(3) | 170 |
| O5w–H5wB···O6w | 0.85 | 2 | 2.756(3) | 148 |

Symmetry codes: (a) ½ – *x,* ½ + *y*, ½ – *z*; (b) *x*, 1 + *y*, *z*; (c) 1 – *x,*1 – *y*, 1 – *z*; (d) 1½ – *x*, -½ + *y*, ½ – *z*; (e) *x*, -1 + *y*, *z*; (f) 1 – *x*,1 – *y*, -*z*; (g) ½ + *x*, ½ – *y*, ½ + *z*; (h) -½ + *x*, 1½ – *y*, -½ + *z*; (i) ½ + *x*, 1½ – *y*, ½ + *z*; (j) ½ + *x*, 2½ – *y*, ½ + *z*.

Table S5. Hydrogen bonds lengths () and angles (°) in [Cu_2_(ida)_2_(2-mim)_2_]_n_·nH_2_O (**4**).

| Donor–H…Acceptor | D–H | H…A | D…A | D–H…A |
| --- | --- | --- | --- | --- |
| N1–H1···O1w | 1.00 | 2.33 | 3.190(5) | 144 |
| N1–H1···O5 | 1.00 | 2.46 | 3.335(5) | 146 |
| N1–H1···O6 | 1.00 | 2.58 | 2.987(5) | 104 |
| O1w–H1wA···O5 | 0.85 | 1.95 | 2.757(4) | 158 |
| O1w–H1wB···O7a | 0.85 | 1.94 | 2.788(4) | 176 |
| N3–H3···O1b | 0.88 | 2 | 2.877(4) | 174 |
| N4–H4···O3c | 1.00 | 1.86 | 2.800(4) | 156 |
| N6–H6···O1wd | 0.88 | 1.91 | 2.773(5) | 167 |

Symmetry codes: (a) -1 + *x, y, z*; (b) ½ – *x*, -½ + *y*, 1½ – *z*; (c) *x*, 1 + *y*, *z*; (d) 1 – *x*, 1 – *y*, 1 – *z*.

Table S6. Selected bond lengths (Å) and angles (º) for [Cu(ida)(2-mim)(H_2_O)_2_]·H_2_O (**1**).

| **1** |  |  |  |
| --- | --- | --- | --- |
| Cu(1)–O(1) | 1.983(2) | Cu(1)–O(2w) | 2.755(2) |
| Cu(1)–O(3) | 1.986 (2) | Cu(1)–N(1) | 1.998(2) |
| Cu(1)–O(1w) | 2.404 (2) | Cu(1)–N(2) | 1.955(2) |
|  |  |  |  |
| O(1)–Cu(1)–O(3) | 163.90(7) | N(1)–Cu(1)–O(1w) | 91.01(7) |
| O(1)–Cu(1)–O(1w) | 91.95 (7) | C(1)–O(1)–Cu(1) | 115.40(14) |
| O(1)–Cu(1)–N(1) | 83.23(7) | C(3)–O(3)–Cu(1) | 113.98(15) |
| O(3)–Cu(1)–O(1w) | 96.86 (7) | C(5)–N(2)–Cu(1) | 128.97(16) |
| O(3)–Cu(1)–N(1) | 83.16(7) | C(5)–N(2)–C(7) | 106.50(2) |
| N(2)–Cu(1)–O(1) | 93.35(8) | C(7)–N(2)–Cu(1) | 123.75(17) |
| N(2)–Cu(1)–O(3) | 99.35(8) | C(2)–N(1)–Cu(1) | 108.12(14) |
| N(2)–Cu(1)–O(1w) | 94.42(8) | C(4)–N(1)–Cu(1) | 107.96 (14) |
| N(2)–Cu(1)–N(1) | 173.69(8) |  |  |

Table S7. Selected bond lengths (Å) and angles (º) for [Cu(ida)(2-mim)_2_]·2H_2_O (**2**).

| **2** |  |  |  |
| --- | --- | --- | --- |
| Cu(1)–O(1) | 2.300(2) | Cu(1)–N(1) | 2.042(2) |
| Cu(1)–O(3) | 1.962(2) | Cu(1)–N(2) | 1.982(2) |
|  |  | Cu(1)–N(3) | 1.988(2) |
|  |  |  |  |
| O(3)–Cu(1)–O(1) | 97.95(7) | N(3)–Cu(1)–N(1) | 92.10(7) |
| O(3)–Cu(1)–N(1) | 84.22(7) | C(3)–O(3)–Cu(1) | 115.91(14) |
| O(3)–Cu(1)–N(2) | 91.51(7) | C(1)–O(1)–Cu(1) | 108.21(13) |
| O(3)–Cu(1)–N(3) | 162.69(8) | C(2)–N(1)–Cu(1) | 108.37(14) |
| N(1)–Cu(1)–O(1) | 76.43(7) | C(4)–N(1)–Cu(1) | 107.90(13) |
| N(2)–Cu(1)–O(1) | 106.16(8) | C(5)–N(2)–Cu(1) | 129.03 (17) |
| N(2)–Cu(1)–N(1) | 175.31(8) | C(7)–N(2)–Cu(1) | 124.26(17) |
| N(2)–Cu(1)–N(3) | 91.42(8) | C(9)–N(3)–Cu(1) | 127.28 (16) |
| N(3)–Cu(1)–O(1) | 97.60(7) | C(11)–N(3)–Cu(1) | 126.17(15) |

Table S8. Selected bond lengths (Å) and angles (º) for [Cu(ida)(2-mim)(H_2_O)]_n_·4.5nH_2_O (**3**).

| **3** |  |  |  |
| --- | --- | --- | --- |
| Cu(1)–O(1) | 1.995(2) | Cu(1)–O(1w) | 2.501(2) |
| Cu(1)–O(3) | 1.999(2) | Cu(1)–N(1) | 1.991(2) |
| Cu(1)–O(2a) | 2.460(2) | Cu(1)–N(2) | 1.953(2) |
|  |  |  |  |
| O(1)–Cu(1)–O(3) | 164.54(6) | N(2)–Cu(1)–O(3) | 99.14(7) |
| N(1)–Cu(1)–O(1) | 83.05(7) | N(2)–Cu(1)–N(1) | 177.26(8) |
| N(1)–Cu(1)–O(3) | 82.77(7) | C(1)–O(1)–Cu(1) | 114.07(14) |
| N(2)–Cu(1)–O(1) | 95.26(7) | C(3)–O(3)–Cu(1) | 113.99(14) |

Table S9. Selected bond lengths (Å) and angles (º) for [Cu_2_(ida)_2_(2-mim)_2_]_n_·nH_2_O (**4**).

| **4** |  |  |  |
| --- | --- | --- | --- |
| Cu(1)–O(3) | 1.990(3) | Cu(2)–O(4) | 2.684(3) |
| Cu(1)–O(1) | 1.992(3) | Cu(2)–O(2a) | 2.945(3) |
| Cu(1)–O(6) | 2.350(3) | Cu(1)–N(2) | 1.965(3) |
| Cu(1)–O(8a) | 2.606(3) | Cu(1)–N(1) | 1.974(3) |
| Cu(2)–O(5) | 1.962(3) | Cu(2)–N(4) | 1.965(3) |
| Cu(2)–O(7) | 1.967(3) | Cu(2)–N(5) | 1.942(3) |
|  |  |  |  |
| O(3)–Cu(1)–O(1) | 165. 45(12) | N(5)–Cu(2)–N(4) | 178.12(15) |
| O(3)–Cu(1)–O(6) | 103.29(12) | C(3)–O(3)–Cu(1) | 113.6(3) |
| O(1)–Cu(1)–O(6) | 82.18(12) | C(9)–O(5)–Cu(2) | 112.9(3) |
| N(2)–Cu(1)–O(3) | 92.42(13) | C(1)–O(1)–Cu(1) | 113.8(3) |
| N(2)–Cu(1)–O(1) | 100.93(13) | C(11)–O(7)–Cu(2) | 114.3(3) |
| N(2)–Cu(1)–O(6) | 92.04 (13) | C(12)–N(4)–Cu(2) | 109.0(3) |
| N(2)–Cu(1)–N(1) | 174.00(15) | C(12)–N(4)–C(10) | 116.9(3) |
| N(1)–Cu(1)–O(3) | 82.08(13) | C(10)–N(4)–Cu(2) | 108.7(2) |
| N(1)–Cu(1)–O(1) | 84.80(13) | C(7)–N(2)–Cu(1) | 122.1(3) |
| N(1)–Cu(1)–O(6) | 86.94(13) | C(9)–O(6)–Cu(1) | 130.7(3) |
| O(5)–Cu(2)–O(7) | 162.31(12) | C(13)–N(5)–Cu(2) | 128.9(3) |
| O(5)–Cu(2)–N(4) | 85.18(13) | C(15)–N(5)–Cu(2) | 125.2(3) |
| N(4)–Cu(2)–O(7) | 84.49(13) | C(2)–N(1)–Cu(1) | 109.5(3) |
| N(5)–Cu(2)–O(5) | 94.79(14) | C(4)–N(1)–Cu(1) | 108.9(2) |
| N(5)–Cu(2)–O(7) | 96.00(14) |  |  |

Table S10. Bond valence calculations for **1** ~ **4**.

| Cu^2+^ | Complexes | Cu1 | Cu2 |
| --- | --- | --- | --- |
| +2 | [Cu(ida)(2-mim)(H_2_O)_2_]·H_2_O (**1**) | 2.106 |  |
| +2 | [Cu(ida)(2-mim)_2_]·2H_2_O (**2**) | 2.173 |  |
| +2 | [Cu(ida)(2-mim)(H_2_O)]_n_·4.5nH_2_O (**3**) | 2.179 |  |
| +2 | [Cu_2_(ida)_2_(2-mim)_2_]_n_·nH_2_O (**4**) | 2.213 | 2.082 |

Table S12. Detailed adsorption data of **4** for O_2_, N_2_, H_2_, CO_2_, CH_4_ and desorption data for O_2_.

| Gases | O_2_ | | O_2_ | | N_2_ | | H_2_ | | CO_2_ | | CH_4_ | |
| --- | --- | --- | --- | --- | --- | --- | --- | --- | --- | --- | --- | --- |
| Temperature(^o^C) | Pressure  (bar) | Adsorption (mg/g) | Pressure  (bar) | Desorption (mg/g) | Pressure  (bar) | Adsorption (mg/g) | Pressure  (bar) | Adsorption (mg/g) | Pressure  (bar) | Adsorption  (mg/g) | Pressure  (bar) | Adsorption  (mg/g) |
| 25.0 | 0 | 0 | 29.897 | 11.799 | 0 | 0 | 0 | 0 | 0 | 0 | 0 | 0 |
|  | 1.890 | 0.908 | 27.895 | 11.038 | 1.900 | 0.162 | 1.901 | 0.068 | 1.900 | 0.366 | 1.890 | 0.070 |
|  | 3.890 | 1.680 | 25.893 | 10.256 | 3.897 | 0.140 | 3.908 | 0.072 | 3.900 | 0.617 | 3.900 | 0.124 |
|  | 5.898 | 2.454 | 23.901 | 9.493 | 5.899 | 0.0470 | 5.910 | 0.057 | 5.897 | 0.675 | 5.899 | 0.144 |
|  | 7.899 | 3.266 | 21.917 | 8.771 | 7.895 | 0.007 | 7.903 | 0.071 | 7.893 | 0.747 | 7.897 | 0.200 |
|  | 9.895 | 4.005 | 19.940 | 8.014 | 9.895 | -0.004 | 9.906 | 0.049 | 9.894 | 0.821 | 9.897 | 0.188 |
|  | 11.895 | 4.818 | 17.958 | 7.215 | 11.896 | 0.003 | 11.901 | 0.074 | 11.894 | 0.802 | 11.894 | 0.155 |
|  | 13.896 | 5.634 | 15.978 | 6.447 | 13.899 | -0.043 | 13.907 | 0.072 | 13.894 | 0.943 | 13.897 | 0.169 |
|  | 15.894 | 6.397 | 13.988 | 5.647 | 15.895 | -0.076 | 15.904 | 0.030 | 15.895 | 0.963 | 15.896 | 0.262 |
|  | 17.896 | 7.176 | 12.003 | 4.848 | 17.897 | -0.025 | 17.905 | 0.065 | 17.893 | 0.987 | 17.897 | 0.177 |
|  | 19.897 | 7.932 | 10.017 | 4.079 | 19.895 | -0.058 | 19.905 | 0.055 | 19.895 | 0.967 | 19.899 | 0.248 |
|  | 21.896 | 8.734 | 8.027 | 3.339 | 21.895 | -0.161 | 21.902 | 0.057 | 21.893 | 0.908 | 21.898 | 0.267 |
|  | 23.896 | 9.424 | 6.035 | 2.586 | 23.896 | -0.202 | 23.908 | 0.051 | 23.891 | 0.924 | 23.899 | 0.242 |
|  | 25.895 | 10.264 | 4.040 | 1.791 | 25.895 | -0.239 | 25.903 | 0.065 | 25.891 | 0.979 | 25.895 | 0.211 |
|  | 27.894 | 11.033 | 2.040 | 0.969 | 27.895 | -0.308 | 27.902 | 0.051 | 27.892 | 0.922 | 27.895 | 0.244 |
|  | 29.897 | 11.799 | 0.010 | 0.156 | 29.894 | -0.336 | 29.906 | 0.032 | 29.893 | 0.854 | 29.894 | 0.221 |
